# ChiCMaxima: a robust and simple pipeline for detection and visualization of chromatin looping in Capture Hi-C

**DOI:** 10.1186/s13059-019-1706-3

**Published:** 2019-05-22

**Authors:** Yousra Ben Zouari, Anne M. Molitor, Natalia Sikorska, Vera Pancaldi, Tom Sexton

**Affiliations:** 1 Institute of Genetics and Molecular and Cellular Biology (IGBMC), Illkirch, France; 2 CNRS UMR7104, Illkirch, France; 3 INSERM U1258, Illkirch, France; 4 University of Strasbourg, Illkirch, France; 5 Centre de Recherches en Cancérologie de Toulouse (CRCT), INSERM U1037, Toulouse, France; 6 University Paul Sabatier III, Toulouse, France; 7 Barcelona Supercomputing Center, Barcelona, Spain

**Keywords:** Promoter-enhancer interactions, Chromatin loops, Capture Hi-C, Biological replicates, Gene regulation, Chromatin assortativity

## Abstract

Capture Hi-C (CHi-C) is a new technique for assessing genome organization based on chromosome conformation capture coupled to oligonucleotide capture of regions of interest, such as gene promoters. Chromatin loop detection is challenging because existing Hi-C/4C-like tools, which make different assumptions about the technical biases presented, are often unsuitable. We describe a new approach, ChiCMaxima, which uses local maxima combined with limited filtering to detect DNA looping interactions, integrating information from biological replicates. ChiCMaxima shows more stringency and robustness compared to previously developed tools. The tool includes a GUI browser for flexible visualization of CHi-C profiles alongside epigenomic tracks.

**Electronic supplementary material:**

The online version of this article (10.1186/s13059-019-1706-3) contains supplementary material, which is available to authorized users.

## Background

The advent of the chromosome conformation capture (3C) technology [1] allowed higher-order chromosome folding to be inferred by identifying spatial proximity between distal genomic sequences, leading to a comprehensive insight of genome topology. As sequencing throughput has increased, it has become feasible to globally assess all chromatin interactions within a population (4C: “one-to-all”; 5C: “many-to-many”; Hi-C: “all-to-all” methods) simply by sequencing all 3C ligation products or a selected subset of them [2–5]. In fact, Hi-C interaction maps can give insight into chromosome folding at different scales, depending on the sequencing depth (and hence resolution) of the study [6, 7]. However, the strength of Hi-C in assessing all possible chromatin interactions is also one of its major disadvantages: the numbers of possible ligation products that can be detected is much greater than the current sequencing output. Recently, several groups have coupled Hi-C (or another 3C derivative) to sequence capture with pools of oligonucleotides complementary to thousands of restriction fragment ends [8–12]. Such “CHi-C” (Capture Hi-C) methods allow the simultaneous and higher resolution mapping of chromatin interactions for large subsets of the genome, such as all promoters or DNase hypersensitive sites. For example, promoter-centered interactomes have already been used to assign epigenomic status and follow enhancer looping dynamics throughout development, as well as to characterize disease-linked intergenic sequence polymorphisms [13–17]. Despite being highly informative, CHi-C datasets have specific properties that set them apart from other 3C-like techniques, which require specialized analytical tools to take these aspects into account. The majority of CHi-C strategies involve large numbers (thousands) of genomically dispersed baits for which interacting regions are detected. The asymmetry between the number of baits and the number of detected interacting regions leads to an asymmetry of CHi-C contact matrices, confounding standard Hi-C normalization approaches. In addition, individual baits have variable capture efficiencies which introduce additional technical biases. Depending on the bait design, CHi-C datasets will be more or less populated with ligation products between two bait fragments (“double-captured” interactions), as well as between bait and non-bait (“single-captured”), which may complicate bias assessment even further.

As for all genome-wide datasets, the challenges for CHi-C analysis are in the appropriate definition of an expected background level, from which “significant” signal can be resolved, and in the development of correct normalization strategies to reduce the impact of non-biological biases. Up to now, three major methods have been described for CHi-C analysis: GOTHiC [18], HiCapTools [19], and CHiCAGO [20]. GOTHiC, actually developed for interaction calling in Hi-C, employs a very simplistic binomial test coupled with multiple testing correction to search for overrepresented interactions, but does not explicitly take into account known features of Hi-C data, such as the heavy dependence of “background” interactions on genomic distance, let alone aspects of CHi-C such as capture bias. HiCapTools uses a substantial portion of negative control probes within the design to better estimate “background” chromosome folding behavior, over which specific looping events can be calculated. However, sufficient numbers of controls are rarely included in many CHi-C applications, limiting the widespread use of this method. CHiCAGO uses a statistical background model to account for different biases in promoter-CHi-C data, combining three factors to define the expected background interaction level: genomic distance, bait capture efficiency, and technical biases present in Hi-C and sequencing approaches [20]. These parameters are fitted to the data to define an expected interaction strength for each individual restriction fragment, based on a combined negative binomial and Poisson variable. However, the treatment of each single fragment as an independent variable creates problems when accounting for biological replicates, since despite its improved coverage compared to Hi-C, current depths of CHi-C datasets still vastly sub-sample the possible space of ligation products. As a result, many reproducible chromatin loops observed at the resolution of larger bins of pooled restriction fragments are lost when scoring individual restriction fragments (Additional file 1: Figure S1). Related to this, it also follows that chromatin interactions comprising contiguous fragments of increased signal, centered on an interaction peak, are less likely to result from technical artifacts than isolated “spikes” of CHi-C signal. CHiCAGO utilizes the same geometric mean approach as DESeq2 [21] to allow weighting for different read depths of different replicates, but this may not completely counter the problem, especially if there is a large discrepancy in numbers of sequence reads between replicates. We tried to overcome these existing limitations of CHi-C analysis methods and developed ChiCMaxima, which we applied to multiple published promoter CHi-C datasets, including mouse embryonic stem (mES) cells with different restriction enzyme and probe design strategies [10, 11], and nine different human primary hematopoietic cell types [13]. Benchmarking against GOTHiC and CHiCAGO consistently showed that ChiCMaxima was a more stringent method for interaction calling, but more robust to handling undersampling when comparing biological replicates. Further, ChiCMaxima gave a higher enrichment for interactions containing hallmarks of regulatory chromatin, such as histone modifications indicative of enhancers or CTCF binding sites, suggesting that its false positive detection rate for functional chromatin loops may be lower than for the other methods. Analysis of the chromatin contact network resulting from ChiCMaxima-called interactions in mES cells identified potential key roles of Polycomb proteins and elongating RNA polymerase II, in line with previous findings [22], further demonstrating the utility of ChiCMaxima. In addition to the pipeline for calling CHi-C interactions, we also present ChiCBrowser, a user-friendly and flexible browser for inputting whole CHi-C datasets and then normalizing and visualizing bait-specific interaction profiles. Tracks of annotated genes and linear epigenomic profiles can also be added to the browser, and called interactions (whether by ChiCMaxima or other methods) can also be highlighted. This tool, whether used standalone or in parallel with ChiCMaxima interaction calling, will aid the community to analyze CHi-C datasets and inform new hypotheses.

## Results

### Methodological foundation of ChiCMaxima

#### Calling interactions as signal local maxima

**Fig. 1:**
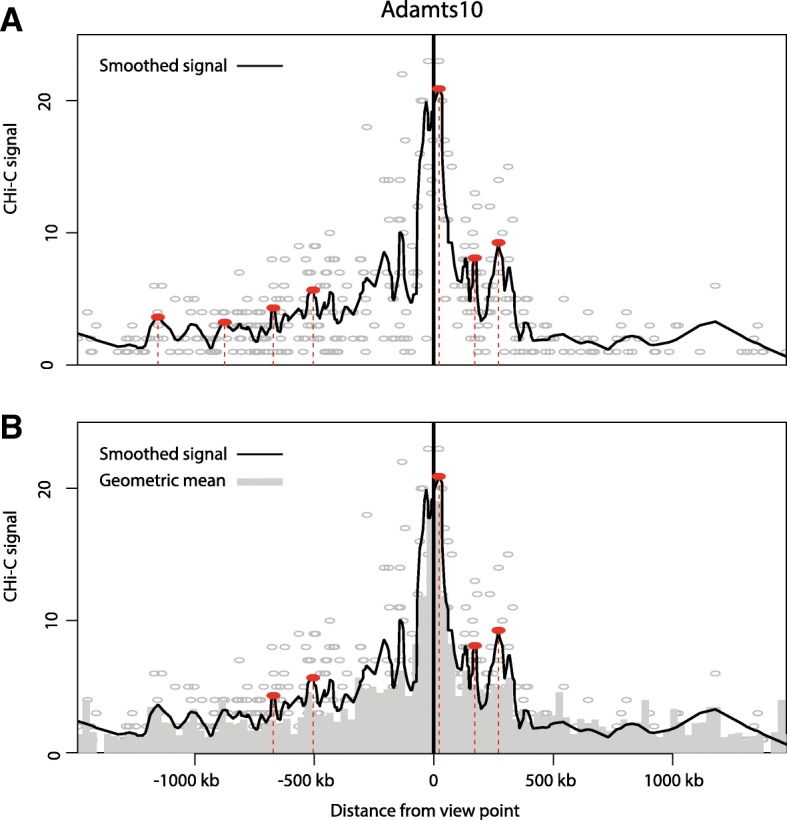
Interaction calling by ChiCMaxima. **a** Virtual 4C profile derived from one mES CHi-C replicate centered on the bait *Adamts10* promoter. The numbers of raw CHi-C sequence reads are plotted as gray circles against their genomic location, and the black line shows the loess-smoothed profile (span = 0.05). Red dotted lines and filled circles denote the positions of called interactions, defined as local maxima of smoothed signal within a fixed number of covered restriction fragments (window = 20). **b** The same virtual 4C profile as **a**, plotted with a bar chart of the geometric means for sequence reads from the profile, stratified by genomic distance between the bait and interacting region (bins of 30 kb). Red dotted lines and filled circles denote the same local maxima as **a**, which have smoothed signal greater than the geometric mean for the corresponding interaction distance, and so are kept as ChiCMaxima-called interactions

In 3C approaches, genomic distance has an important impact on the expected frequency of interactions. Generally, the frequency of interactions decays with a power law as the genomic distance between fragments increases, consistent with many polymer physics models [4]. DNA loops correspond to a peak of higher interaction signal compared to the expected level of neighbor fragments on either side; this principle was used to detect loops in some of the first 3C studies [23]. To detect peaks in the signal, we use a naïve, non-parametric approach to call local maxima, making limited prior assumptions about the data (Fig. 1). The theoretical basis and proof of principle of ChiCMaxima is presented below; an operational guide and breakdown of the pipeline’s different tools is detailed in Additional file 2.

First, treating each bait independently and removing bait-to-bait and inter-chromosomal interactions, we obtain a “virtual 4C” profile of read counts relative to the genomic position of the non-bait fragment and perform loess smoothing on this profile. The fragments with the maximum signal are identified within sliding windows of a fixed fragment number, and local maxima are defined as regions where the smoothed signal equals this value. With this approach, only three parameters need to be controlled: the span of the loess smoothing (*s*), the window size (*w*) for the local maximum computation, and the total genomic span (*c*, for *cis-distance from bait*) over which local maxima are assessed. Over-smoothing or using too large window size may cause some maxima to be missed, and under-smoothing or small window sizes may call many local spikes as spurious interactions. We performed a parameter sweep for local maxima calling on a subset of mES promoter CHi-C data (all covered pairwise contacts (~ 1.5 million) for 2000 randomly sampled baits within a single biological replicate). We found that the numbers of interactions called most heavily depended on the *w* parameter (Additional file 1: Figure S2a, b; see Additional file 3 for the full exploration of ChiCMaxima parameters). As expected, larger *w* provided the greatest stringency, calling fewer maxima which were supported by greater numbers of sequencing reads. However, large *w* also placed a heavy bias on calling the shortest-range interactions, potentially precluding detection of the functional chromatin loops that are known to take place over megabase scales [24–26]. Changes to the *s* or *c* parameters made relatively little difference to interaction calling (see Additional file 3). Regardless of the choice of ChiCMaxima parameters, we observed that local maxima with very low signal, often very distant from the bait (and thus with a negligible background signal from neighboring fragments), are still called as “interactions” (Fig. 1a). We thus opted to remove these spurious calls with additional filtering.

#### Bait-specific filtering

According to previous work on CHi-C data [20], the background interaction level at short genomic distances (up to ~ 1.5 Mb) is largely dominated by genomic separation (proposed to be caused by Brownian collisions of the chromosome fiber). In CHiCAGO, a cubic-fitted log-distance function was derived from the geometric means of read counts for binned genomic separations and was then scaled with capture bias estimates in the final derived background distribution [20]. Inspired by this, we searched for similar but *bait-specific* approaches to apply to each virtual 4C profile. The advantage of this approach is that the data from different baits, which may reside in very different chromatin environments, do not need to be pooled together. The major limitation is that the relative paucity of bait-specific data could lead to overfitting in the model, particularly if a strong interaction causes overestimation of “background” signal around it. Indeed, we found that cubic or linear fits of bait-specific log-distance functions performed poorly—very few called maxima were filtered out as having fewer supporting reads than the background estimate (Additional file 1: Figure S2c, d). Instead, we noted better removal of spurious weak interactions by simply filtering out called maxima with fewer supporting reads than the geometric mean for bait-specific contacts within the corresponding genomic separation bin (Fig. 1b). Importantly, this filter allowed us to improve the confidence of called interactions using smaller maximum-calling windows (*w* parameter), maintaining stringency while reducing the bias for shorter-range interactions. This approach was robust to different widths of the genomic separation bins, *b*, used for computing geometric means (see Additional file 3 for full details). The major limitation of this approach is that it only serves to remove spuriously called local maxima and does not provide a meaningful background model of “expected” contacts. As a result, ChiCMaxima calls interactions without giving a measure of interaction strength. Based on our parameter sweep, we opted for the following parameters for the majority of subsequent analyses: *w* 50 fragments; *s* 0.05; *c* 1.5 Mb; *b* 30 kb.

#### Accounting for biological replicates

Although CHi-C improves on the resolution afforded by conventional Hi-C, it remains an under-sampled method. Although taking the intersection of called interactions from all replicates will give the highest-confidence chromatin loops, the false negative rate appears to be very high from this approach, due to poor reproducibility at the single restriction fragment level, both for CHiCAGO and for the better-performing ChiCMaxima (Additional file 1: Figure S1). We noted that many interaction peaks from one biological replicate also had adjacent or very close peaks in the second replicate, even though they were not at exactly the same restriction fragment (Additional file 1: Figure S3a). To see if these are likely to represent the same biological interactions, we assessed more systematically the distributions of genomic distance between interacting regions called in one biological replicate and the closest interaction called in the second replicate of the mES CHi-C data (Additional file 1: Figure S3b). Indeed, around one fifth of ChiCMaxima-called interactions had no genomic separation across replicates, meaning that they were on the same or directly contiguous restriction fragment, and more than a third of all interactions were found within 20 kb (~ 5 *Hind*III restriction fragments), suggesting that genomic interactions called by CHi-C can indeed be reproducibly called across replicates, albeit at a lower resolution than single restriction fragments. To add more flexibility for analyzing biological replicates, ChiCMaxima allows a threshold distance between reported peaks in biological replicates to be defined by the user (*d*: default in the tool is 0). After local maximum computation and filtering on each biological replicate, these interactions are further filtered to retain only those where an interaction is also called within distance *d* in all other biological replicates. Unless stated otherwise, CHi-C analysis in this manuscript is performed with the parameter *d* = 20 kb. ChiCMaxima also provides a tool for assessing the distributions of closest distances between interactions called in pairs of biological replicates, better informing the user on their choice of the *d* parameter (see Additional file 2 for details).

### Benchmarking of ChiCMaxima

**Fig. 2:**
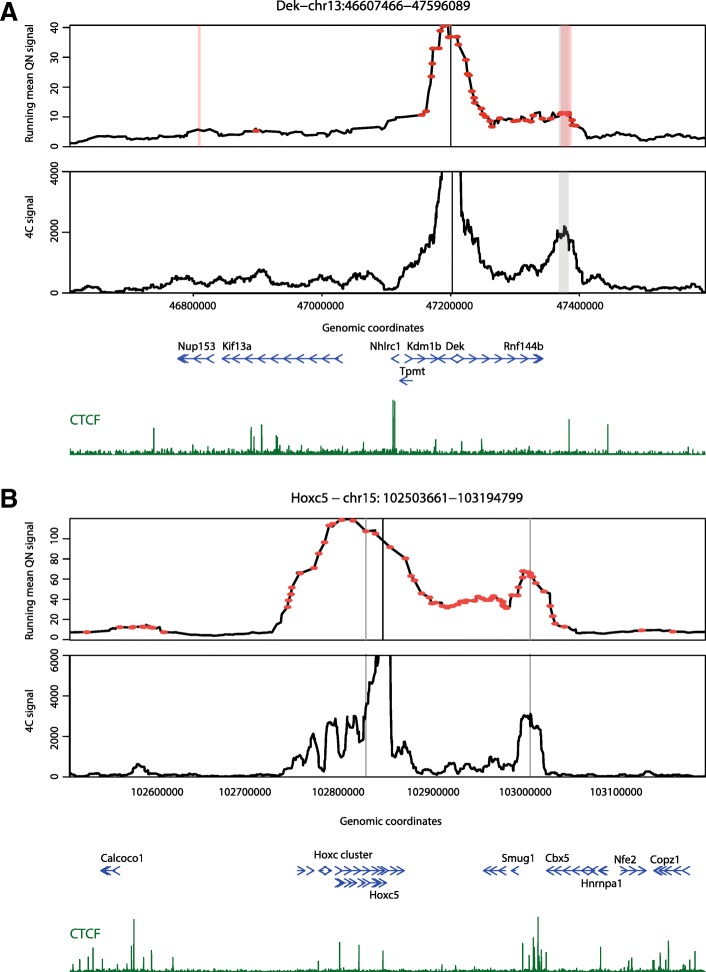
ChiCMaxima precisely calls chromatin interactions. **a** mES CHi-C (upper panel) and 4C (lower panel) profiles centered on the bait *Dek* promoter are shown. The interactions called by ChiCMaxima and CHiCAGO are denoted as stripes (gray and pink, respectively); points denote interactions called by GOTHiC. GOTHiC seemingly calls many spurious interactions. **b** mES CHi-C (upper panel) and 4C (lower panel) profiles centered on the bait *Hoxc5* promoter are shown. The interactions called by ChiCMaxima are denoted as gray stripes, and a large number of seemingly spurious interactions called by CHiCAGO are denoted as red points. Called interactions conserved between ChiCMaxima and CHiCAGO are centered on CTCF sites. For both profiles, gene position (blue) and CTCF ChIP-seq profiles (dark green) are shown below the CHi-C and 4C profiles

**Fig. 3:**
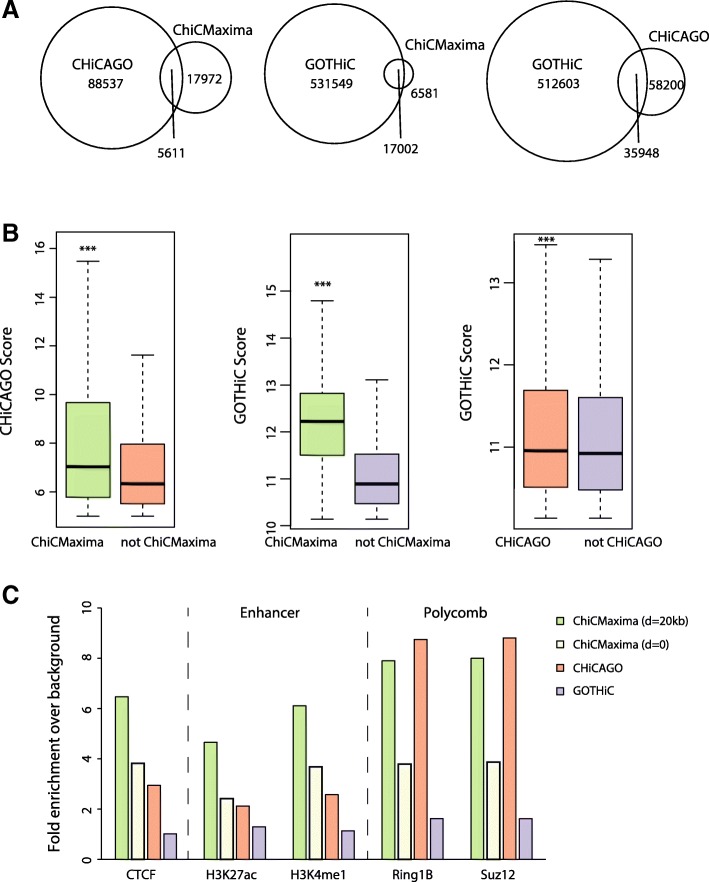
Comparison of ChiCMaxima, CHiCAGO, and GOTHiC on mES CHi-C data. **a** Venn diagrams showing numbers of interactions called by the three different methods which are conserved with the other methods. **b** Box plots comparing the CHiCAGO (left) or GOTHiC (center and right) metric scores of interaction strength for the sets of interactions called by CHiCAGO (left) or GOTHiC (center and right) which are conserved with those called by ChiCMaxima (left and center) or CHiCAGO (right), versus those which are not. ***P < 2 × 10^−16^; Wilcoxon rank sum test. **c** Bar charts showing fold enrichment over genomic background for different ChIP-seq peaks within the promoter-interacting sequences determined by the different CHi-C analysis methods

We performed ChiCMaxima on a published mES promoter CHi-C dataset [11] and compared our results with published ones from GOTHiC and CHiCAGO applied to the same dataset [11, 20] (Table 1; Additional file 4: Table S1). On visual inspection, ChiCMaxima successfully identified clear promoter interactions, some of which we also validated by 4C, and seemed to call fewer spurious ones than the other two methods (Fig. 2). Indeed, ChiCMaxima identified fewer promoter-centered interactions (23,583) than CHiCAGO (94,148) or GOTHiC (548,551). Pairwise comparisons revealed a striking dissimilarity of called interactions across all three methods—with the exception of ChiCMaxima interactions within the GOTHiC set, the majority of called interactions from one method is not shared with those of another (Fig. 3a). This is likely due to the very different assumptions made in the models for each method. We next sought to compare the performance of each method in calling chromatin interactions that are most likely to be functionally relevant, and minimizing likely false positives. First, we tested the hypothesis that ChiCMaxima, in calling fewer interactions than the other two methods, was the most stringent tool, calling only higher-confidence interactions. We split the interaction sets called by CHiCAGO or GOTHiC into those that were recapitulated, or not, by ChiCMaxima. In both cases, the interactions maintained in ChiCMaxima had significantly higher metrics of interaction score (weighted probability score in CHiCAGO [20]; observed/expected ratio in GOTHiC [18]) than for interactions called by the other method alone (Fig. 3b; *P* < 2 × 10^−16^, Wilcoxon rank sum test). Interactions conserved by CHiCAGO and GOTHiC calling also had significantly higher observed/expected ratios than interactions called in GOTHiC alone, but with a much more modest effect size. We thus conclude that ChiCMaxima is indeed the most stringent of the CHi-C interaction calling methods, calling the higher-confidence interactions of the other methods.

**Table 1:** Overview of CHi-C interactions called by CHiCAGO, GOTHiC, and CHiCMaxima

	CHiCAGO [20]	GOTHiC [11]	ChiCMaxima (this manuscript)	ChiCMaxima and CHiCAGO
Number of called interactions	94,148	548,551	23,583	5611
Mean number of called interactions per bait	4.2	29.4	1.4	0.34

#### Epigenomic analysis of ChiCMaxima-called interactions

One of the major perceived applications of CHi-C is to assign target genes to candidate *cis*-regulatory elements, particularly enhancers, by virtue of the specific interactions they make with promoters. Genomic studies revealed that enhancers share hallmark chromatin features: monomethylation of histone H3 lysine-4 (H3K4me1), DNase-hypersensitivity, acetylation of histone H3 lysine-27 (H3K27ac), and/or p300 co-activator occupancy [27]. However, despite epigenomic predictions of enhancers in numerous cell types, unambiguous identification of their target genes has proved more elusive, since they can control multiple genes and may skip one or several promoters to act over large distances [28]. Promoter CHi-C studies have indeed shown a general enrichment in interacting regions bearing enhancer chromatin signatures [8, 10, 11], as well as for regions bound by CTCF, a known factor implicated in chromatin loops [29]. We reasoned that an interaction calling method that found the greatest proportion of putative enhancers and/or CTCF sites within a promoter CHi-C dataset was most likely to have the best true positive detection rate. Based on this, ChiCMaxima compares favorably to the other two methods. It has a higher enrichment for interacting regions containing CTCF, H3K27ac, and H3K4me1 (Fig. 3c), with a ~ 2-fold improvement over CHiCAGO and ~ 5-fold improvement over GOTHiC. The enrichment in these functional hallmarks is decreased when the *d* parameter of ChiCMaxima is reduced to zero, but is still slightly better than CHiCAGO. Chromatin interaction networks mediated by Polycomb group proteins have also been well described in embryonic stem cells [17, 22, 30, 31]. Reflecting this, promoter-interacting regions called by ChiCMaxima and CHiCAGO are also comparably and highly enriched in binding for core components of the two major Polycomb repressive complexes, Ring1B and Suz12 (Fig. 3c). Importantly, ChiCMaxima has a consistently higher enrichment for interacting regions containing CTCF and enhancer marks when different ChiCMaxima caller parameters are used (increasing or decreasing *w*; increasing *c*), further demonstrating the robustness of the tool (Additional file 1: Figure S4a and Table S2). Since more than half of ChiCMaxima-called interactions are not conserved with CHiCAGO, we also asked whether combining both methods would improve predictive power further. Indeed, the enrichment in functional hallmarks is even higher within the 5611 interactions that are conserved in both tools (Additional file 1: Figure S4b), indicating that combining the two methods gives the most stringent, highest-confidence interactions that are the most likely to be functionally relevant. However, the high enrichment for functional marks within ChiCMaxima-alone (and to a lesser extent for enhancer marks, CHiCAGO-alone) interactions implies that many functional interactions are also likely to be missed by intersecting the two methods. This is also apparent on visual inspection of called interactions within CHi-C profiles (Additional file 1: Figure S4c).

Additionally, we assessed which of the 19,200 candidate mES enhancers (based on chromatin signatures [32]) could be assigned to target promoters by the different methods (Table 2; Additional file 1: Table S3). As expected, the proportion of assigned enhancers scaled with the numbers of total called interactions (71.4% for GOTHiC, 19.2% for CHiCAGO, 16.8% for ChiCMaxima). However, candidate enhancers comprised a much higher proportion of the ChiCMaxima-called interaction set than for the other two methods (~ 3-fold higher than CHiCAGO; ~ 5-fold higher than GOTHiC), in line with the relative enrichments for individual regulatory marks. The interactions called by both ChiCMaxima and CHiCAGO only assign target genes to 4.8% of putative enhancers, with a modest increase in proportions of putative enhancers within the interaction set. Interaction sets called by ChiCMaxima with different parameters contained very similar proportions of candidate enhancers (Additional file 1: Table S3).

**Table 2:** Overview of putative mES enhancers found within CHi-C interactions called by different methods

	Putative mES enhancers in called interaction set	Total called interactions/interactions with putative enhancers
ChiCMaxima	16.8% (3235)	7.3
CHiCAGO	19.2% (3680)	25.6
GOTHiC	71.4% (13711)	40.0
ChiCMaxima + CHiCAGO	4.8% (930)	6.0

#### ChiCMaxima performance in other CHi-C datasets

**Fig. 4:**
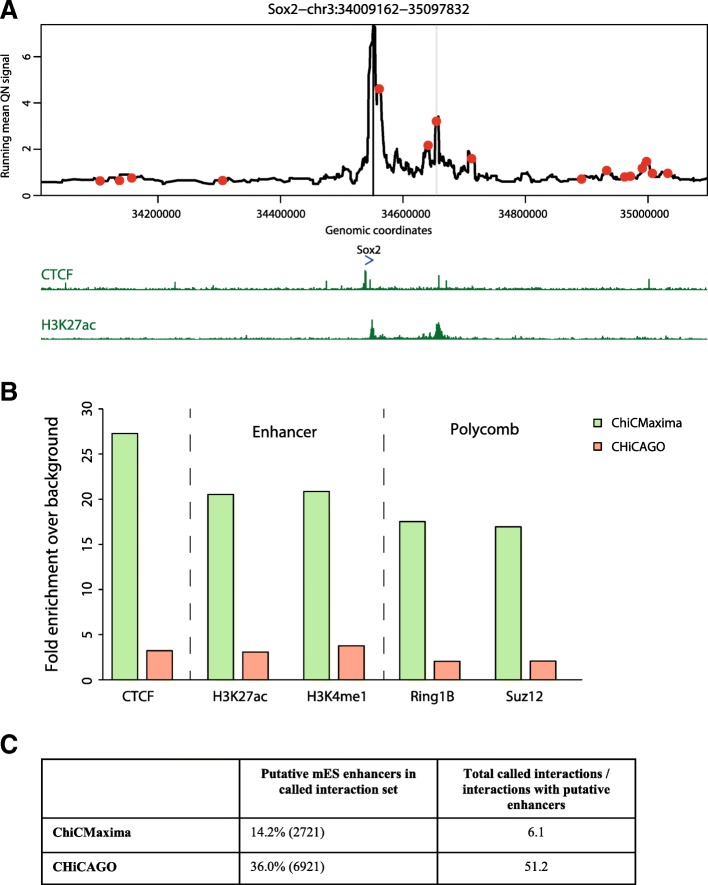
ChiCMaxima performance on mES HiCap data. **a** mES HiCap profile centered on the *Sox2* promoter. The interaction with the *Sox2* enhancer called by ChiCMaxima is denoted as a gray stripe, and CHiCAGO-called interactions, including a large number of seemingly spurious ones, are denoted as red points. Gene position (blue) and selected mES ChIP-seq profiles (dark green) are shown below the HiCap profile. **b** Bar charts showing fold enrichment over genomic background for different ChIP-seq peaks within the promoter-interacting sequences determined by ChiCMaxima and CHiCAGO. **c** Overview of putative mES enhancers found within HiCap interactions called by ChiCMaxima and CHiCAGO

To test whether the tuned ChiCMaxima parameters are more globally applicable, and to more comprehensively benchmark the method, we applied ChiCMaxima with the standard parameters (*w* = 20, *s* = 0.05, *b* = 30 kb, *c* = 1.5 Mb, geometric mean filter) to other published CHi-C datasets. Notably, whereas ChiCMaxima and CHiCAGO were readily applied to these data, we were unable to implement GOTHiC due to the very high memory requirement of the method. First, we called interactions from a complementary mES promoter CHi-C dataset (two biological replicates), which used a different probe design and a more frequently cutting restriction enzyme, *Mbo*I, in the HiCap strategy [10]. These present an analytical challenge, since they have been relatively less deeply sequenced, and are derived from a much more complex mixture of Hi-C ligation products (~ 200-fold more possible pairwise restriction fragment combinations). Despite this greater complexity, the same proportion of HiCap interactions were reproduced across biological replicates within *d* = 20 kb as for CHi-C (~ 40%). Perhaps due to its reliance on calling interactions at the level of single restriction fragments within a more complex pool of products, CHiCAGO called ~ 5-fold more interactions from the HiCap data than from CHi-C; ChiCMaxima actually called 1.5-fold fewer interactions (Additional file 5: Table S4). However, visual inspection of the different calls on HiCap profiles, assessment of CTCF, enhancer and Polycomb mark enrichments, and the proportions of candidate enhancers contained within the interaction sets strongly indicate that ChiCMaxima is the more stringent, robust interaction calling method (Fig. 4).

**Fig. 5:**
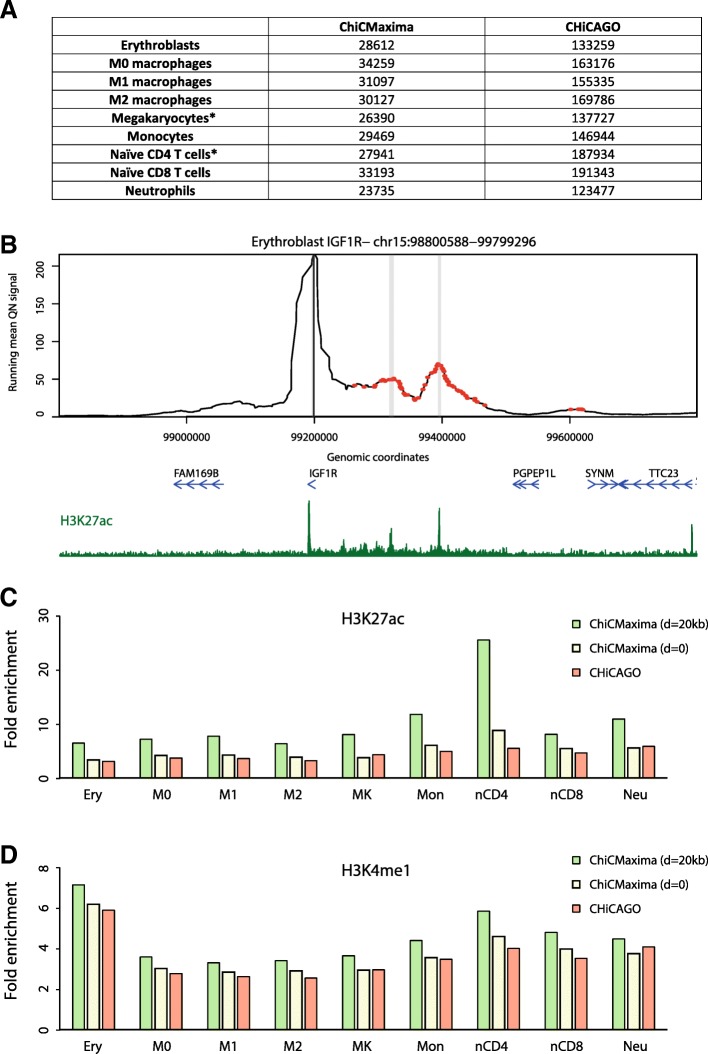
ChiCMaxima performance on CHi-C data from human primary hematopoietic cells. **a** Numbers of interactions called by ChiCMaxima and CHiCAGO on the different datasets (three biological replicates for all, except for those denoted by an asterisk, which had four biological replicates). **b** Erythroblast CHi-C profile centered on the *IGF1R* promoter. Interactions called by ChiCMaxima are denoted as gray stripes, and those called by CHiCAGO are denoted as red points. Gene position (blue) and the erythroblast H3K27ac ChIP-seq profile (dark green) are shown below the CHi-C profile. **c** Bar charts showing fold enrichment over genomic background for H3K27ac peaks within the promoter-interacting sequences determined by ChiCMaxima and CHiCAGO for the nine hematopoietic cell types. **d** Bar charts showing fold enrichment over genomic background for H3K4me1 peaks within the promoter-interacting sequences determined by ChiCMaxima and CHiCAGO for the nine hematopoietic cell types

We then compared ChiCMaxima and CHiCAGO interaction calling within deeply sequenced CHi-C datasets derived from nine different primary human hematopoietic cell types (erythroblasts; M0, M1, and M2 macrophages; megakaryocytes; monocytes; naïve CD4 and CD8 T cells; neutrophils) [13]. An additional test of the analytical methods came from the presence of greater numbers of biological replicates (3–4). For all pairwise combinations of biological replicates, at least 50% of interactions were maintained within *d* = 20 kb. We found that extending the ChiCMaxima method for handling two replicates (keeping only interactions that are present in both replicates, within a threshold distance, *d* (usually 20 kb), of each other) to three or four performed well (see Additional file 2 for operational details). Strictly requiring that interactions are present within a fixed window of *d* for all of the replicates called similar numbers of interactions as for the two replicates of mES CHi-C; furthermore, applying this method to the datasets with four biological replicates (megakaryocytes and naïve CD4 T cells) did not drastically reduce the numbers of called interactions as compared to those with three replicates (Fig. 5a; Additional file 6: Table S5, Additional file 7: Table S6, Additional file 8: Table S7, Additional file 9: Table S8, Additional file 10: Table S9, Additional file 11: Table S10, Additional file 12: Table S11, Additional file 13: Table S12, Additional file 14: Table S13). As previously, ChiCMaxima more stringently calls fewer, higher-confidence interactions, which are apparent on visual inspection of CHi-C profiles (Fig. 5b and Additional file 1: Figure S5), and consistently gave higher enrichment for putative enhancers, marked by H3K27ac and H3K4me1 (Fig. 5c, d). Stricter filtering among replicates, setting *d* to 0, gave similar epigenomic enrichments to CHiCAGO, with ChiCMaxima nearly always performing slightly better.

Although there is not a large overlap between interactions called by the two methods (Fig. 3a; Additional file 1: Figure S4c), the possibility remains that ChiCMaxima does not call interactions fundamentally differently to CHiCAGO and just sets a higher threshold than the default CHiCAGO score. To formally test this, we also compared epigenomic enrichments between interacting regions called by ChiCMaxima, and the matched number of interactions with the highest scores in CHiCAGO (Additional file 1: Figure S6). The CHiCAGO threshold scores were not identical in the different cell types, but were much higher than the standard threshold of 5. Importantly, ChiCMaxima gave consistently higher enrichments for H3K27ac and, to a lesser extent, H3K4me1, suggesting that the method does more than simply modulate the threshold of existing interaction calling tools. Overall, these results suggest that ChiCMaxima provides a good compromise of stringency and coverage when assigning target genes to putative *cis*-regulatory elements, with a robust set of parameters that can be globally applied to different CHi-C datasets.

### Chromatin assortativity analysis comparing CHiCAGO and ChiCMaxima derived contact networks

**Fig. 6:**
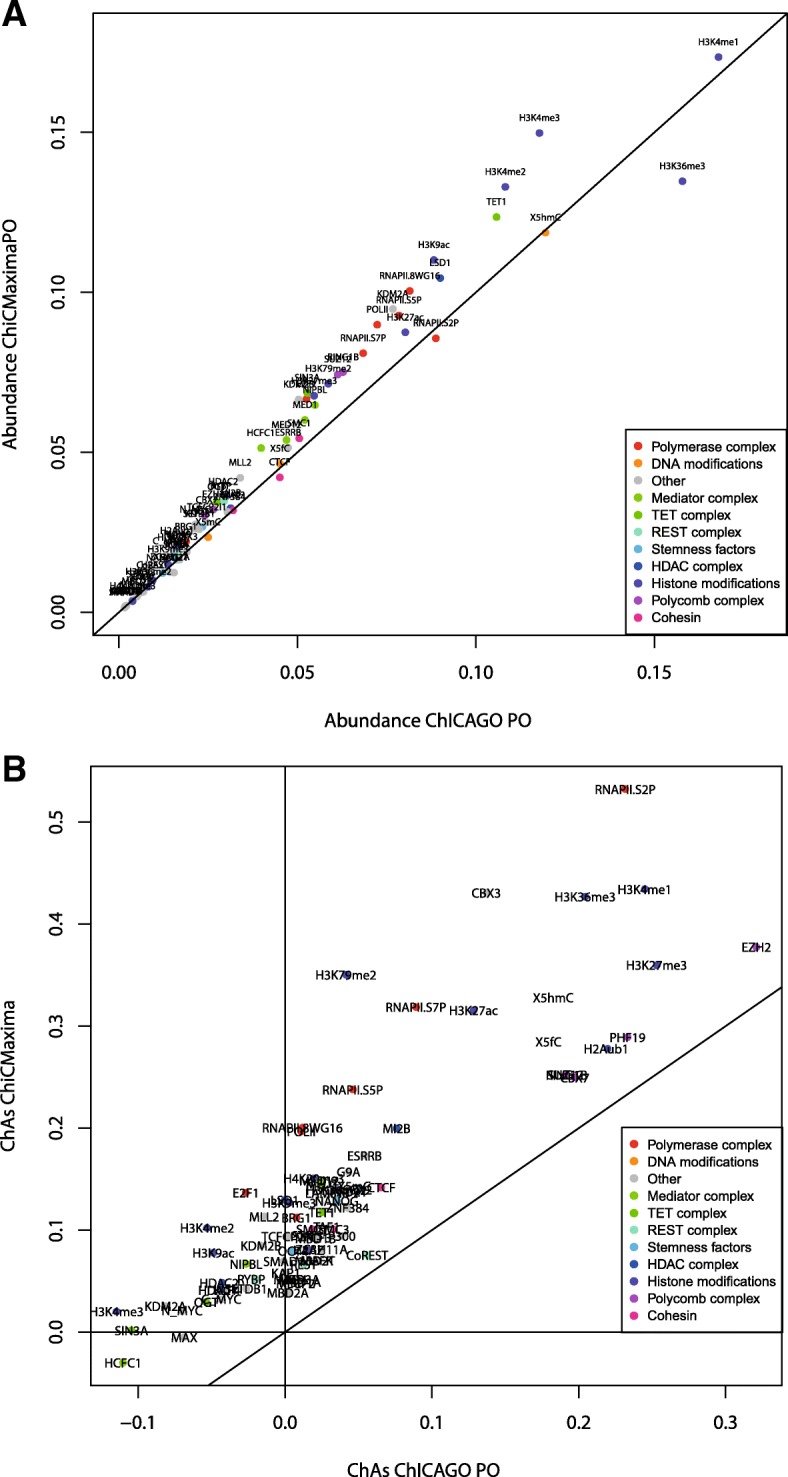
Exploration of chromatin assortativity of different features on the ChiCMaxima-generated chromatin contacts derived from the mES CHi-C data. **a** Scatter plot of abundance of different chromatin features within the interaction networks called by ChiCMaxima or CHiCAGO. **b** Scatter plot of chromatin assortativity of different chromatin features within the interaction networks called by ChiCMaxima or CHiCAGO (restricted to promoter-other end interactions). The class of the different chromatin features is color coded

Despite great progress in the experimental mapping of chromatin organization inside the nucleus, many questions regarding the functional impact of its structure remain unanswered. It is thus difficult to estimate the accuracy of any interaction calling algorithm beyond the performance on the few regions of the genome that are well characterized. Moreover, alongside the known role of interactions in bringing enhancer regions close to their target genes and grouping Polycomb-repressed genes, there might be other functionally relevant 3D chromatin structures which we still do not understand, hence the need for finding complementary analytical methods to study the panorama of genome-wide interactions. A recent step in this direction was made by looking at chromatin contact maps as networks and applying methods from network theory to gain a comprehensive understanding of nuclear organization (e.g., [22, 33–35]). For example, appreciation of the chromatin interaction network topology bolstered the link between spatial gene co-associations and their co-expression patterns [33]. An important concept that was recently applied to chromatin interaction networks is *assortativity*—which indicates the extent to which genomic regions sharing the same chromatin mark(s) preferentially interact. This property is not trivially related to the relative abundance of a mark at interacting regions, and highly assortative chromatin features are more likely to be related to chromatin interactions. A recent study of mES chromatin interactions identified three major chromatin features that were highly assortative: the abundant H3K4me1 mark, features of transcriptional elongation (predominantly RNA polymerase II phosphorylated on serine-2 of the C-terminal repeat domain (RNAPII-S2P) and trimethylation of lysine-36 of histone H3 (H3K36me3)), and the relatively low abundance Polycomb group proteins and associated histone marks (e.g., trimethylation of lysine-27 of histone H3 (H3K27me3) [22]. To further test the utility of ChiCMaxima, we applied chromatin assortativity (ChAs) analysis to the network of ChiCMaxima-called interactions and directly compared it to the one derived by CHiCAGO for promoter-other end interactions (Fig. 6; Additional file 1: Figure S7). Although the relative abundances of the different chromatin features were very similar (Pearson correlation coefficient 0.98), and the three aforementioned categories of assortative chromatin features were identified by the two methods (Pearson correlation coefficient 0.87 for ChAs values obtained on the two networks), some differences were apparent. In particular, transcriptional elongation hallmarks are very strongly flagged by ChiCMaxima. In addition to RNAPII-S2P and H3K36me3, other features enriched within active gene bodies in ES cells, such as dimethylation of histone H3 lysine-79 (H3K79me2) and CBX3 (HP1γ; associated with transcriptional elongation and stem cell identity [36, 37]), were also revealed to be highly assortative by ChiCMaxima. These results demonstrate that ChiCMaxima-called interactions can be used in informative network analyses and highlight promoter-gene body contacts as a potentially important architectural feature for active genes (see the “Discussion” section).

### ChiCBrowser

**Fig. 7:**
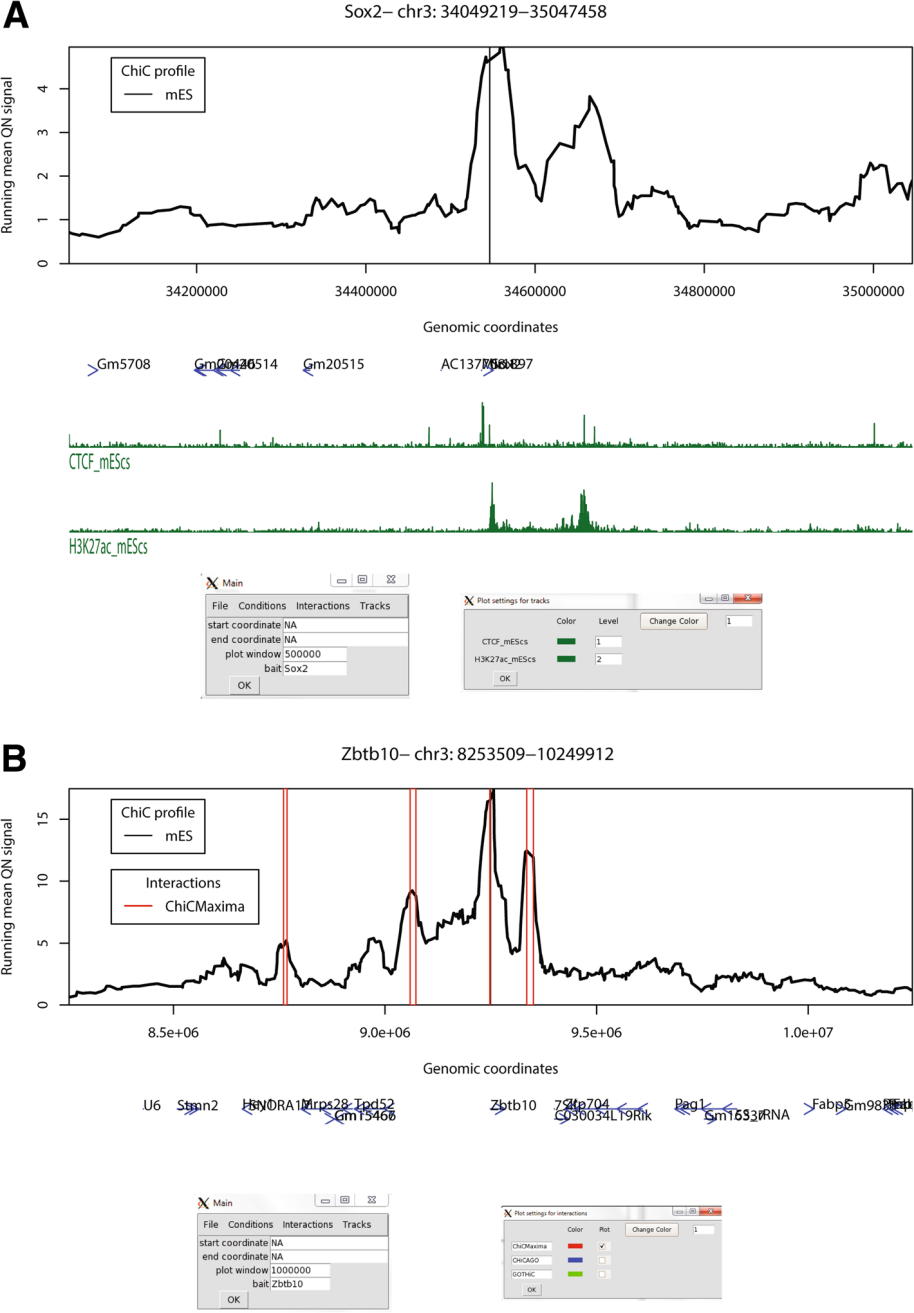
Some functionalities of ChiCBrowser. **a** A screenshot of ChiCBrowser, showing the mES CHi-C profile for 500 kb up- and downstream of the bait *Sox2* promoter. Gene positions (blue) and selected mES ChIP-seq tracks (green) are shown below the profile. The main ChiCBrowser user interface window is shown underneath (left), where the bait and plot window have been specified. A sub-window, called from the *Tracks* menu (right), allows the color and level of the epigenomic profiles to be controlled by the user. Epigenomic tracks that are given the same level (for instance, the same histone mark in different tissues) are scaled to the same level on the *y*-axis so that the profiles are visually comparable. **b** As for **a**, a screenshot of the mES CHi-C profile for 1 Mb up- and downstream of the bait *Zbtb10* promoter. Open red rectangles show the position of interactions called by ChiCMaxima. The sub-menu on the bottom right, called from the *Interactions* menu, allows the user to control which interaction lists to annotate on the CHi-C plot

To enable visualization of promoter (and other sparse bait) CHi-C results, alongside linear epigenomic profiles and the interactions called by ChiCMaxima or other methods, we also developed ChiCBrowser, an R-based GUI browser. Unlike the WashU browser [38], which displays all interactions simultaneously and can be difficult to interpret visually, ChiCBrowser displays virtual 4C profiles, with the bait and display window defined by the user via a graphical window (Fig. 7). Its major functionalities are described below; a full user guide is given in Additional file 2.

All CHi-C datasets which may be plotted together or compared are made into one input file (see Additional file 2 for input format details), which only needs to be loaded once into the memory for all subsequent plots to be made. To allow fairer comparisons between datasets, all CHi-C-derived virtual 4C profiles are quantile normalized [39] before the running mean values are plotted. Since CHi-C experimental designs usually include biological replicates and different conditions to be compared, ChiCBrowser provides flexibility (via the *Conditions* menu) to define the plot levels of each single CHi-C dataset. For example (shown in Additional file 1: Figure S8), biological replicates can be allocated to different levels and plotted side by side to compare experimental reproducibility, or given the same plot level, so that the mean profile can be plotted for comparison with other experimental conditions. The user can assign names to these plot levels and change their plotting colors.

The *Tracks* menu allows the user to load gene annotations (as a modified bed file; see Additional file 2), which are plotted as blue arrows to show transcriptional orientation, and linear epigenomic profiles in bigWig or bedGraph formats. Similar to the *Conditions*, the user can define plot levels for epigenomic profiles (Fig. 7a). In this case, this defines which profiles are scaled to the same level on the *y*-axis, for instance allowing fairer comparison between profiles of the same histone mark mapped in different tissue types. The epigenomic profile plot colors can also be modified by the user.

Ostensibly, the *Interactions* menu allows the user to load sets of interactions called by ChiCMaxima (or CHiCAGO, whose output is in the same format) for them to be highlighted on the CHi-C profile (Fig. 7b). However, the input format of these interactions is essentially the chromosomal coordinates of genomic regions associated with a specific bait (see Additional file 2 for details), so this plotting functionality can be adapted to highlight any subset of the CHi-C dataset that the user designs (e.g., interactions unique to one condition or tissue type and not another). This flexibility in particular makes ChiCBrowser very useful to explore different hypotheses when browsing interactomes. As for other ChiCBrowser functions, the user can alter the name and color of these annotations, as well as select or de-select subsets of them.

## Discussion

We present two tools for processing and interpretation of Chi-C datasets: ChiCMaxima for interaction calling and ChiCBrowser for bait-specific visualization of interaction profiles. Both were developed to overcome the currently identified unique challenges presented by these data. Despite a clear improvement over conventional Hi-C with limited sequencing throughput, the main issue with CHi-C outputs is that they are greatly under-sampled, creating problems of reproducibility across biological replicates at the highest resolutions (Additional file 1: Figure S1 and S3). The subsequent paucity of bait-specific data confounds the generation of powerful statistical models, so previous methods either appear to have high false positive rates (e.g., GOTHiC; see Fig. 2), and/or rely on combining data from multiple baits (e.g., CHiCAGO) to avoid overfitting model parameters. ChiCMaxima uses a limited number of model parameters to be estimated by naively just searching for local maxima in the virtual 4C profiles (Fig. 1), a logic for calling chromatin loops that was used in some of the first 3C studies [23, 40]. Application of an additional filter was necessary to remove spurious local maxima in distal regions of low signal, and we found satisfactory results from a simple threshold based on geometric means of reads stratified by interaction distance (Additional file 1: Figure S2; Additional file 3). For single datasets, only four parameters need to be defined in ChiCMaxima: the window for local maximum computation, the loess smoothing span, the total genomic span over which maxima are computed, and the bin width for stratifying the geometric mean filter. Of these, interaction calling is only very sensitive to the local maximum computation window (Additional file 3), and in any case, we found improved performance over existing methods for a wide range of parameters (Additional file 1: Figure S4a), indicating that ChiCMaxima is fairly robust to parameter choice. A major advantage of ChiCMaxima is thus that interactions can simply be called without the need to control or estimate multiple parameters, or choose arbitrary thresholds. However, this advantage also means that ChiCMaxima does not return measurements of statistical significance interpretable as the interaction “strength” of the called chromatin loops. When comparing chromatin interactions between different tissues or conditions, we find that quantile normalization allows fair visual comparisons (e.g., Additional file 1: Figure S1 and S3), but further work will be required to better define and quantify interaction strength differences.

As mentioned previously, another major challenge resulting from the undersampling of CHi-C data is the handling of biological replicates. Presumably because it processes sliding windows rather than treating each restriction fragment independently, ChiCMaxima has superior reproducibility to CHiCAGO, but this is still less than 10% at the single restriction fragment level (Additional file 1: Figure S1). Since many interactions are reproduced at slightly lower resolutions (Additional file 1: Figure S3), ChiCMaxima has a built-in flexibility whereby interactions can be filtered for those that are conserved in all replicates, within a user-defined distance. The optimal distance may be expected to vary between experiments, particularly with sequencing depth and complexity of the assessed genome. For this reason, we provide tools to allow the user to explore the distributions of closest distances between interactions called in pairs of replicates and thus determine the optimal setting. However, for all 11 CHi-C datasets we tested, these distributions were very similar, with ~ 40% of interactions within 20 kb of each other in replicate experiments. We also note that such an approach is robust to inclusion of more than two biological replicates and that ChiCMaxima performance was still better than other methods at the most restrictive condition of allowing no distance between reproduced interactions within replicates (e.g., Figs. 3 and 5), albeit with reduced sensitivity.

Despite the simplistic approach of ChiCMaxima, it compares favorably to GOTHiC and CHiCAGO in various different benchmarks in different CHi-C experimental setups, suggesting that it is one of the more stringent calling methods (thus likely reducing false positives) to successfully call a high proportion of interactions that are likely to be functionally relevant (Figs. 3, 4, and 5). This includes tests of the following: reproducibility between biological replicates; increased metrics of interaction strength within ChiCMaxima-called interactions; enrichment for putative enhancer marks, CTCF binding sites, and Polycomb-bound regions within promoter-interacting regions; assignment of putative enhancers to target genes; proportion of putative enhancers within the called interaction set; reduced apparent false positive rate on visual inspection of CHi-C profiles (e.g., Fig. 2). We note that promoter interactions with non-enhancer/CTCF/Polycomb-bound elements may certainly be frequent and functionally significant, albeit poorly characterized so far. Indeed, all three methods call many interactions of this category. However, the greater enrichment of ChiCMaxima-called interactions for promoter-enhancer loops that have been so well described in the literature, coupled with their overall higher interaction score metrics as called by other methods, suggests that ChiCMaxima is the most stringent interaction calling method, but also reliably identifies interactions most likely to be functionally relevant. However, the apparent inconsistency in interaction calls between the three methods (Fig. 3a), coupled with the good enrichment for regulatory marks in CHiCAGO-only interactions, suggests that ChiCMaxima has some false negatives which are correctly detected by CHiCAGO (the inverse also seems to be the case). Indeed, the highest-confidence interactions are conserved between CHiCAGO and ChiCMaxima, but the false negative rate seems very high when relying on this stringent approach. Although we present multiple lines of evidence suggesting that ChiCMaxima has a lower false positive rate (higher *specificity*) than previous methods, it is much more difficult to assess if and to what extent ChiCMaxima may have an increased false negative rate (reduced *sensitivity*), failing to call “true” interactions. Visual inspection of CHi-C profiles shows many cases where ChiCMaxima failed to call an apparently real interaction that was found by CHiCAGO and vice versa, but in the absence of comprehensive prior knowledge of the promoter interactome, we are unable to quantify the methods’ sensitivities. Overall, we recommend using ChiCMaxima when looking for global features of chromatin interactions, since the false positive rate seems lower, but combinations of ChiCMaxima and CHiCAGO may be required to comprehensively explore the interactomes of specific baits of interest. We also note that ChiCMaxima, due to its dependence on searching for local maxima, is not suitable for assessing ultra-long-range or *trans* interactions, where the background signal is too sparse for local maxima to even be called. Bait-to-bait interactions should also not be assessed by ChiCMaxima, since these double-captured interactions are highly likely to appear as “artificial” local maxima when flanked by single-captured, bait-to-non-bait interactions within sliding windows. Finally, CHi-C strategies using tiled oligonucleotides to intensively cover a contiguous domain [9, 41] are better analyzed with the suite of tools adapted to the contact matrices generated by 5C or Hi-C (e.g., adaptations of my5C [42] or Juicer [43]).

As a further demonstration of the utility of ChiCMaxima, network analysis of called chromatin interactions also identified the Polycomb-mediated interactome that has been previously described in ES cells [17, 22, 30, 31] (Fig. 6). Interestingly, the ChiCMaxima network also indicates frequent contacts between promoters and the bodies of active genes, a phenomenon which was also identified by the same analysis of the CHiCAGO network, but to a lesser extent [22], and was also reported in a recent study assessing multiplex chromatin interactions [44]. It is currently unclear whether this may be an indirect effect of transcriptional elongation on topology of the chromosome fiber [45], or reflects more specific mechanisms of gene expression control. For example, enhancers have been described to initially contact promoters, but to additionally track along the gene during transcriptional elongation [46], and promoter and enhancer interactions with specific exons have been implicated in splicing control [47, 48]. Further studies will be required to determine the functional significance, if any, of such intragenic chromatin looping events, but ChiCMaxima seems to be a very useful tool for studying them via CHi-C studies.

The ChiCBrowser tool is a flexible, user-friendly GUI to generate virtual 4C profiles, necessary for visual inspection of most CHi-C datasets. It has a built-in flexibility to allow biological replicates or different combinations of biological conditions to be assessed in parallel, and a similar flexibility is also built into the management of gene annotations and epigenomic profiles that are plotted alongside the CHi-C data (Fig. 7). Called interactions, whether by ChiCMaxima or other methods, can be easily highlighted on the display, based on a simple input format that can be adapted to highlight any subset of the CHi-C subset that may be of interest to the user. Overall, this browser will be of use to anyone wishing to explore CHi-C data.

## Conclusions

Capture Hi-C, particularly strategies with sparse baits such as promoters, is a rapidly growing technique hampered by the limited tools available to meet the unique challenges of analyzing the datasets produced. ChiCMaxima adopts a simplistic approach, with minimal prior assumptions on the data, and successfully calls CHi-C interactions, performing favorably with existing methods in various benchmarks. Most notably, ChiCMaxima provides the flexibility to deal with problems of reproducibility across biological replicates at high resolutions, a persistent but often overlooked challenge of CHi-C. Combined with the user-friendly, flexible ChiCBrowser, we provide a suite of tools for CHi-C analysis and visualization which will be of use to many in the nuclear organization community.

## Methods

### Datasets used in this study

Mouse ES CHi-C [11] and HiCap [10] data were downloaded from ArrayExpress (E-MTAB-2414) [49] and GEO (GSE60495) [50], respectively; human hematopoietic cell CHi-C data [13] were downloaded as CHiCAGO data objects from the Open Science Framework (https://osf.io/u8tzp/) [51]. Interactions previously called by CHiCAGO and GOTHiC from the mES CHi-C data were downloaded from GEO (GSE81503) [52] and ArrayExpress (E-MTAB-2414) [49] respectively. Mouse ES ChIP-seq data [53–55] were all downloaded from GEO: CTCF and H3K27ac (GSE29218) [56]; H3K4me1 (GSE47082) [57]; Ring1B and Suz12 (GSE42466) [58]. All human hematopoietic cell ChIP-seq data were obtained from the BLUEPRINT consortium (ftp://ftp.ebi.ac.uk/pub/databases/blueprint/data/homo_sapiens/GRCh37/) [59]. The list of putative mES enhancers was taken from Table S1 of Chen et al. [32].

### Sample pre-processing

For all mES (CHi-C and HiCap) datasets, raw sequencing reads were processed by custom perl and R scripts, originally derived from the Hi-C analysis pipeline developed in [60], which entails mapping the paired reads with Bowtie [61], pairing, removing common Hi-C artifacts (PCR duplicates, circularized fragments, non-digested fragments), and then converting from genomic coordinates to restriction fragment space. Operationally, this generates only tiny differences from outputs of HiCUP [62]. Custom perl scripts, explained in Additional file 2 and available on Github [63], were used to convert paired bed files to the input format for ChiCMaxima. These scripts can also be applied to outputs of other Hi-C analysis tools, such as HiCUP [62], HiC-Pro [64], or Juicer pre-inputs [43]. For the human hematopoietic cell CHi-C datasets, total CHiCAGO output files were downloaded as R objects [51]. These comprise the downstream results of HiCUP processing of the data and CHiCAGO analysis (on merged biological replicates), resulting in a table containing all the fields required for ChiCMaxima analysis (see Additional file 2 for details), for all bait-linked interactions covered by sequencing reads. These tables were manipulated in R to make separate tables for each replicate in a format compatible with the ChiCMaxima scripts and to remove interchromosomal and bait-to-bait interactions.

### ChiCMaxima

The suite of scripts, made for R version ≥ 3.2, and its full documentation (including package dependencies, found on Bioconductor or CRAN), is available on Github (https://github.com/yousra291987/ChiCMaxima) [63]. A full description of its usage, and how it is run on supplied test data, is also provided in Additional file 2. In brief, ChiCMaxima_Caller identifies interactions as local maxima of loess smoothed bait-specific interaction profiles within single CHi-C datasets. ChiCMaxima_RepAnalysis determines the distributions of the closest distance between interactions called in pairs of datasets, allowing the user to select an optimal threshold for filtering “maintained” interactions within biological replicates. ChiCMaxima_MergeRep2 or ChiCMaxima_MergeRepMany then applies this set distance threshold to identify interactions that are conserved in two or more biological replicates, respectively. Finally, ChiCMaxima_Collate is a utility script that generates one large table from multiple CHi-C datasets, convenient for input into ChiCBrowser. Except where stated specifically in the text, ChiCMaxima_Caller was run on each single CHi-C replicate with the parameters *window_size* = 20, *loess_span* = 0.05, *cis_window* = 1,500,000, and binwidth = 30,000. ChiCMaxima_MergeRep2 or ChiCMaxima_MergeRepMany was run on their outputs with the parameter *repdist* = 20,000.

### ChiCBrowser

The browser is run from an R environment (version ≥ 3.2), and its full documentation (including package dependencies, found on Bioconductor or CRAN) is also available on Github (https://github.com/yousra291987/ChiCMaxima) [63]. A full user guide is also presented in Additional file 2, along with examples of its use on supplied test data. This browser or small variants in the code (e.g., to show raw data instead of after smoothing by running means in Additional file 1: Figure S1b) were used to generate all the screenshot images presented in the article.

### CHiCAGO and GOTHiC interaction lists

The previously called lists of interactions from both mES CHi-C replicates using CHiCAGO (GSE81503_mESC_PCHiC_merge_final_washU_text.txt; CHiCAGO score ≥ 5) or GOTHiC (ESC_promoter_other_significant_interactions.txt; log (observed/expected) ≥ 10) were downloaded directly from their repositories [49, 52]. CHiCAGO-called interactions from human primary hematopoietic cell CHi-C datasets were downloaded directly from their repository [51], and then filtered to remove interchromosomal and bait-to-bait interactions. For each cell type, the interactions were called as those with a score ≥ 5. For interaction calling within individual biological replicates by CHiCAGO, interactions with a score ≥ 5 were used after running CHiCAGO with default parameters (maxLBrownEst = 1,500,000; minFragLen = 150; maxFragLen = 40,000; minNPerBait = 250; binsize = 20,000; removeAdjacent = TRUE; adjBait2bait = TRUE; tlb.filterTopPercent = 0.01; tlb.minProxOEPerBin = 50,000; tlb.minProxB2BPerBin = 2500; techNoise.minBaitsPerBin = 1000; brownianNoise.samples = 5; brownianNoise.subset = 1000; brownianNoise.seed = NA; weightAlpha = 34.11573; weightBeta = − 2.586881; weightGamma = − 17.13478; weightDelta = − 7.076092). The same parameters were used for CHiCAGO interaction calls on the HiCap data (both replicates treated simultaneously). Intersections of called interactions across biological replicates (Additional file 1: Figure S1a) were found by searches for called interactions with identical Bait_name and ID_OE columns.

### Tuning ChiCMaxima parameters and filter choices

See Additional file 3 for details.

### Assessing distances between potentially conserved interactions across biological replicates

This is performed by ChiCMaxima_RepAnalysis. Pairs of interaction files (the output of ChiCMaxima_Caller) are split according to their bait, and one set is defined as the *query* and the other set as the *subject*. For each non-bait fragment within the query, the genomic distance to the closest non-bait fragment within the subject set is found by the utilities within the R GenomicRanges package [65].

### 4C interaction validation

J1 mouse ES cells were grown on gamma-irradiated mouse embryonic fibroblast cells under standard conditions (4.5 g/L glucose-DMEN, 15% FCS, 0.1 mM non-essential amino acids, 0.1 mM beta-mercaptoethanol, 1 mM glutamine, 500 U/mL LIF, gentamicin), then passaged onto feeder-free 0.2% gelatin-coated plates for at least two passages to remove feeder cells. Cells were detached with trypsin, washed by centrifugation in PBS, and then fixed with 2% formaldehyde in mES culture medium for 10 min at 23 °C. The fixation was quenched with cold glycine at a final concentration of 125 mM, then cells were washed with PBS and permeabilized on ice for 1 h with 10 mM Tris-HCl, pH 8, 100 mM NaCl, 0.1% NP-40, and protease inhibitors. Nuclei were resuspended in *Dpn*II restriction buffer at 10 million nuclei/mL concentration, and 5 million nuclei aliquots were further permeabilized by treatment for 1 h with 0.4% SDS at 37 °C, then a further 1 h with 2.6% Triton-X100 at 37 °C. Nuclei were digested overnight with 1000 U *Dpn*II at 37 °C, then washed twice by centrifuging and resuspending in T4 DNA ligase buffer. In situ ligation was performed in 400 μL T4 DNA ligase buffer with 20,000 U T4 DNA ligase overnight at 16 °C. DNA was purified by reverse cross-linking with an overnight incubation at 65 °C with proteinase K, followed by RNase A digestion, phenol/chloroform extraction, and isopropanol precipitation. The DNA was digested with 5 U/μg *Csp*6I at 37 °C overnight (for *Dek*) or 5 U/μg *Tai*I at 65 °C for 2 h (for *Hoxc5*), then re-purified by phenol/chloroform extraction and isopropanol precipitation. The DNA was then circularized by ligation with 200 U/μg T4 DNA ligase under dilute conditions (5 ng/μL DNA) and purified by phenol/chloroform extraction and isopropanol precipitation. Fifty-nanogram aliquots of this DNA were used as template for PCR with bait-specific primers containing Illumina adapter termini (primer sequences and optimal PCR conditions available on request). PCR reactions were pooled, primers removed by washing with 1.8x AMPure XP beads, then quantified on a Bioanalyzer (Agilent) before sequencing with a HiSeq 4000 (Illumina). Sequence reads were filtered and mapped to *Dpn*II restriction fragments, essentially as previously described [5, 66]. Raw and processed 4C data are available on GEO (GSE129884) [67]. For visualization of the 4C profiles, running means of read counts across windows of 25 restriction fragments are plotted against the genomic coordinate of the fragment interacting with the bait (Fig. 2).

### Comparing CHi-C calling methods

The intersections in interaction calling methods (Fig. 3a) were computed using the R GenomicRanges package [65] to find overlapping coordinates within the non-bait regions from interaction sets with the same bait. Comparisons of the interaction scores from CHiCAGO or GOTHiC-called interactions which were or were not conserved with another method were computed by Wilcoxon rank sum tests.

### Assessing enrichment for epigenomic marks

For mES, ChIP-seq fastq files were aligned to the mm9 genome with bowtie2 [61], then peaks were called with the Erange 4.0 ChIP-seq peak finder tools [68, 69], with the settings --nodirectionality, --notrim and an FDR threshold of 0.05. ChIP-seq peaks for human primary hematopoietic cells were downloaded directly from their repository [59]. Enrichment of each epigenetic feature within an interaction set was computed by dividing the proportion of interactions (non-bait component) overlapping with a feature peak within the interaction set by the proportion of all mappable, non-bait restriction fragments which overlap with a feature peak. These overlaps were found using bedtools [70] on the bed files of non-bait interacting regions versus the bed files of called ChIP-seq peaks. Overlaps of the set of putative mES enhancers [32] with non-bait regions within called interactions were performed with GenomicRanges.

### Chromatin assortativity

Interaction network analysis was performed exactly as described in [22]. Briefly, 78 chromatin features were taken from [71] and peak-calling/binarization was performed as described there in 200-bp windows. For each fragment, the overlapping windows of chromatin peaks were identified and their values averaged to give a fraction of presence of any feature in each fragment. The abundance of a feature is defined as the average of that feature value across all fragments in the network considered. ChAs of a specific chromatin feature is defined as the Pearson correlation coefficient of the value of that feature across all pairs of nodes that are connected with each other. They are computed from the “assortativity” function of the R package igraph. We created a network of ChiCMaxima detected interactions (39,584 nodes, 23,583 edges). Interactions captured by ChiCMaxima were assumed to be all involving a bait and a non-bait (other end) region, but we observed that some of the non-bait fragments captured were overlapping baits (4754 fragments), effectively suggesting that some of the interactions captured are promoter-promoter interactions (6457 interactions involving 3895 promoters). The method is not supposed to capture this type of interactions as shown by the low percentage of contacts that fall in this category and we therefore removed these interactions in the following analysis, which was performed with a network of promoter-other (PO) end interactions (35,207 nodes and 20,100 interactions). ChAs were computed from the total interaction network derived by ChiCMaxima, which omits bait-to-bait interactions. To avoid confounding effects of bait-to-bait interactions present within the full CHiCAGO-called network, ChAs computation was restricted to only the promoter-to-other end (P-O) portion of the network.

## Additional files

Additional file 1: **Figure S1.** Under-sampled CHi-C datasets confound analyses at single restriction fragment level. **Figure S2.** Testing parameters of ChiCMaxima. **Figure S3.** CHi-C interaction calling across biological replicates. **Figure S4.** Epigenomic enrichments from alternative interaction calling methods. **Figure S5.** Improved stringency of ChiCMaxima over CHiCAGO when applied to human primary hematopoietic cell CHi-C data. **Figure S6.** ChiCMaxima is not just a more stringent version of CHiCAGO. **Figure S7.** Scatter plot of chromatin assortativity against relative feature abundance for different chromatin features within the ChiCMaxima-called interaction network derived from the mES CHi-C dataset. **Figure S8.** Flexibility in handling replicates in ChiCBrowser. **Table S2.** Overview of CHi-C interactions called by ChiCMaxima with different parameters. **Table S3.** Overview of putative mES enhancers found within CHi-C interactions called by ChiCMaxima with varying parameters. (PDF 5746 kb)

Additional file 2: ChiCMaxima and ChiCBrowser user guide. (PDF 355 kb)

Additional file 3: Optimizing ChiCMaxima parameters. (PDF 274 kb)

Additional file 4: **Table S1.** List of mES interactions called by ChiCMaxima from two CHi-C replicates. (XLSX 1734 kb)

Additional file 5: **Table S4.** List of mES interactions called by ChiCMaxima from two HiCap replicates. (XLSX 1166 kb)

Additional file 6: **Table S5.** List of human erythroblast interactions called by ChiCMaxima from three CHi-C replicates. (XLSX 2329 kb)

Additional file 7: **Table S6.** List of human M0 macrophage interactions called by ChiCMaxima from three CHi-C replicates. (XLSX 2738 kb)

Additional file 8: **Table S7.** List of human M1 macrophage interactions called by ChiCMaxima from three CHi-C replicates. (XLSX 2503 kb)

Additional file 9: **Table S8.** List of human M2 macrophage interactions called by ChiCMaxima from three CHi-C replicates. (XLSX 2433 kb)

Additional file 10: **Table S9.** List of human megakaryocyte interactions called by ChiCMaxima from four CHi-C replicates. (XLSX 2274 kb)

Additional file 11: **Table S10.** List of human monocyte interactions called by ChiCMaxima from three CHi-C replicates. (XLSX 2381 kb)

Additional file 12: **Table S11.** List of human naïve CD4 T cell interactions called by ChiCMaxima from four CHi-C replicates. (XLSX 2389 kb)

Additional file 13: **Table S12.** List of human naïve CD8 T cell interactions called by ChiCMaxima from three CHi-C replicates. (XLSX 2644 kb)

Additional file 14: **Table S13.** List of human neutrophil interactions called by ChiCMaxima from three CHi-C replicates. (XLSX 1968 kb)
