# Supplementary material for: ChiCMaxima: a robust and simple pipeline for detection and visualization of chromatin looping in Capture Hi-C
Source: Genome Biol. 2019 May 22;20:102. doi: 10.1186/s13059-019-1706-3 (PMC6532271; doi:10.1186/s13059-019-1706-3)
Supplement: Supplementary file 1 — Figure S1. Under-sampled CHi-C datasets confound analyses at single restriction fragment level. Figure S2. Testing parameters of ChiCMaxima. Figure S3. CHi-C interaction calling across biological replicates. Figure S4. Epigenomic enrichments from alternative interaction calling methods. Figure S5. Improved stringency of ChiCMaxima over CHiCAGO when applied to human primary hematopoietic cell CHi-C data. Figure S6. ChiCMaxima is not just a more stringent version of CHiCAGO. Figure S7. Scatter plot of chromatin assortativity against relative feature abundance for different chromatin features within the ChiCMaxima-called interaction network derived from the mES CHi-C dataset. Figure S8. Flexibility in handling replicates in ChiCBrowser. Table S2. Overview of CHi-C interactions called by ChiCMaxima with different parameters. Table S3. Overview of putative mES enhancers found within CHi-C interactions called by ChiCMaxima with varying parameters. (PDF 5746 kb) [file 13059_2019_1706_MOESM1_ESM.pdf]

ChiCMaxima: a robust and simple pipeline for detection and visualization of chromatin looping in Capture Hi-C

Additional File 1: Figures S1-S8, and Tables S2 and S3

Yousra Ben Zouari, Anne M Molitor, Natalia Sikorska, Vera Pancaldi and Tom Sexton

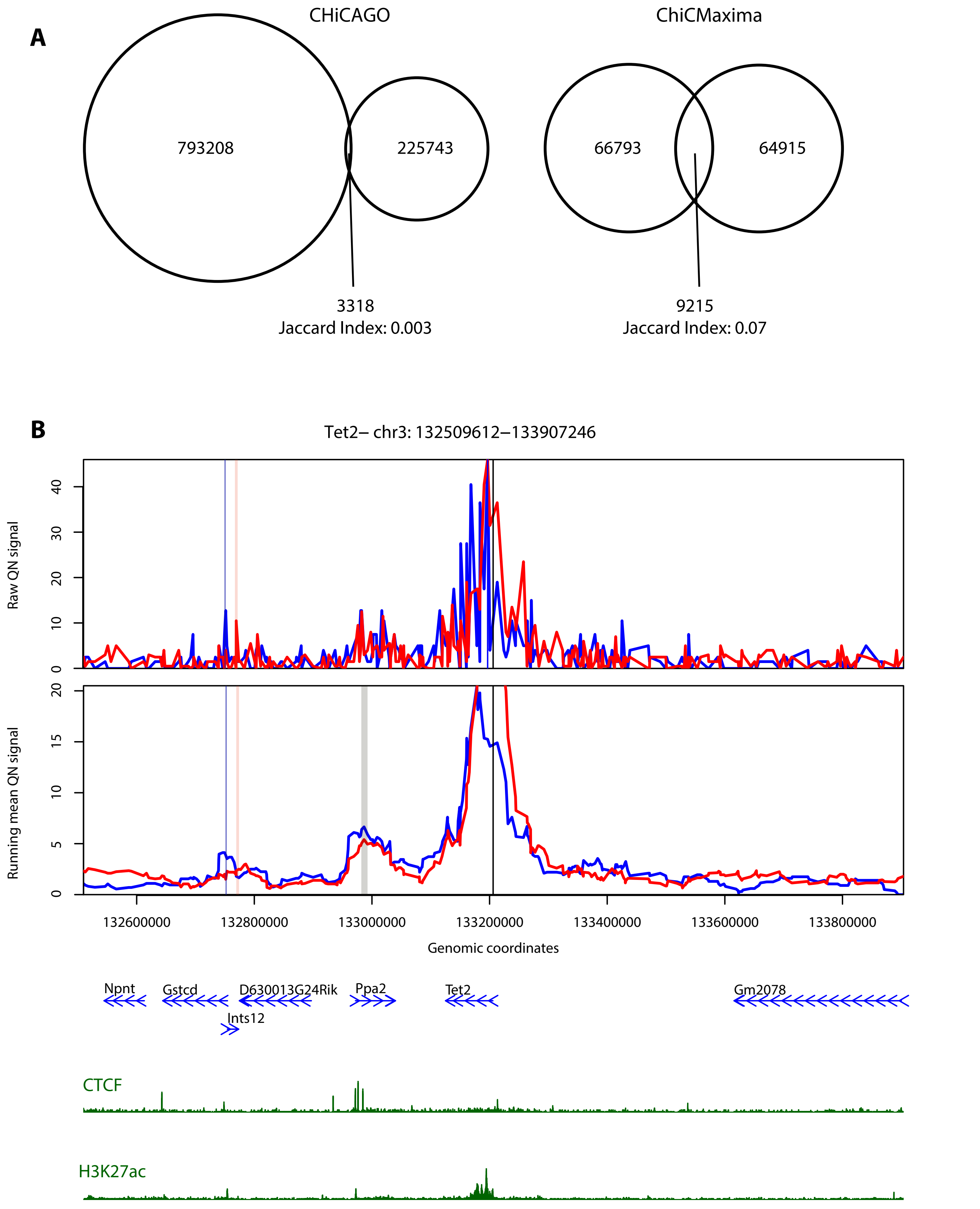
A
CHiCAGO
793208
225743
3318
Jaccard Index: 0.003
ChiCMaxima
66793
64915
9215
Jaccard Index: 0.07
B
Tet2– chr3: 132509612–133907246
Raw QN signal
Running mean QN signal
Genomic coordinates
132600000
132800000
133000000
133200000
133400000
133600000
133800000
Npnt
Gstcd
D630013G24Rik
Ppa2
Tet2
Gm2078
Ints12
CTCF
H3K27ac

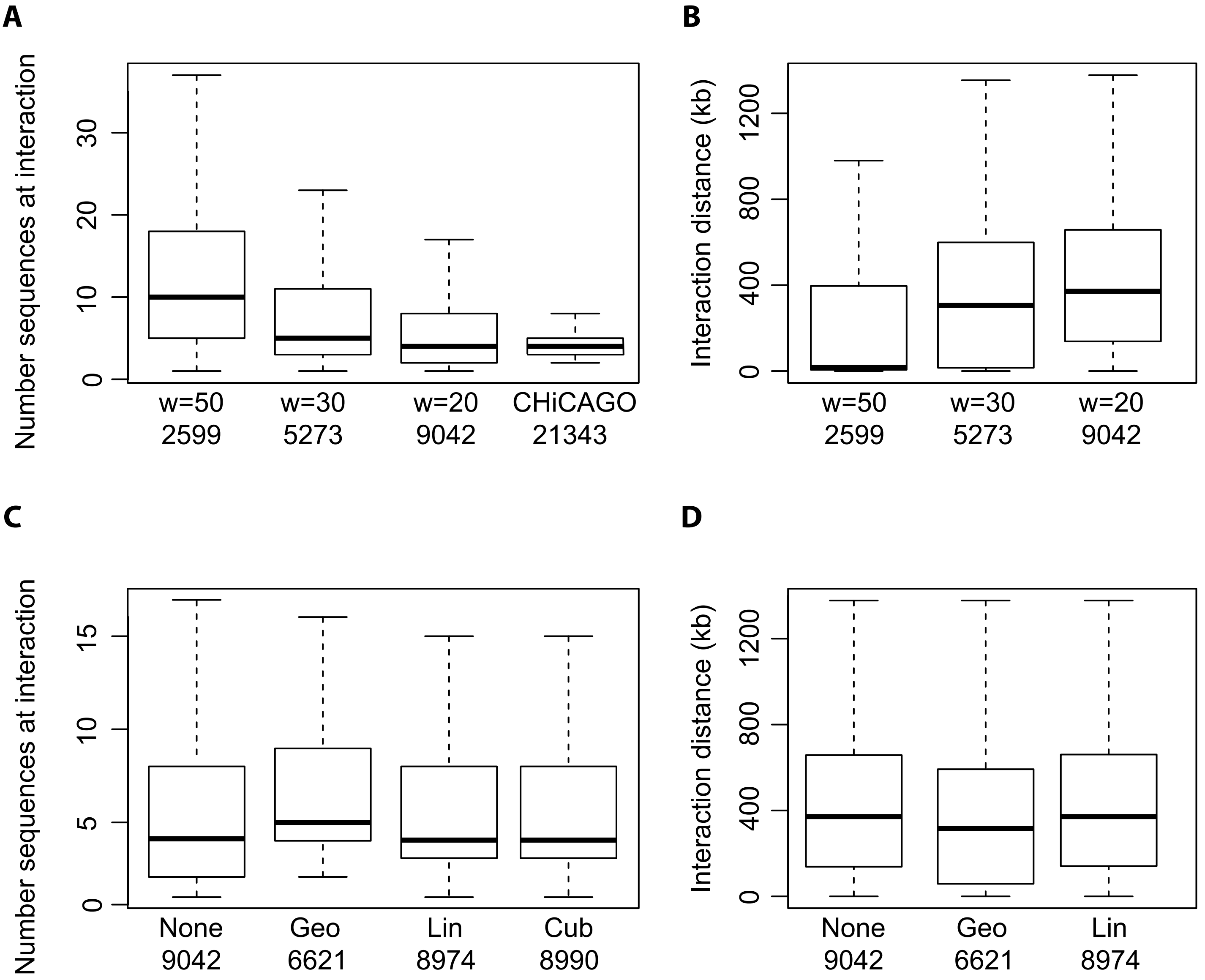
A
Number sequences at interaction
0
10
20
30
w=50
2599
w=30
5273
w=20
9042
CHiCAGO
21343
B
Interaction distance (kb)
0
400
800
1200
w=50
2599
w=30
5273
w=20
9042
C
Number sequences at interaction
0
5
10
15
None
9042
Geo
6621
Lin
8974
Cub
8990
D
Interaction distance (kb)
0
400
800
1200
None
9042
Geo
6621
Lin
8974

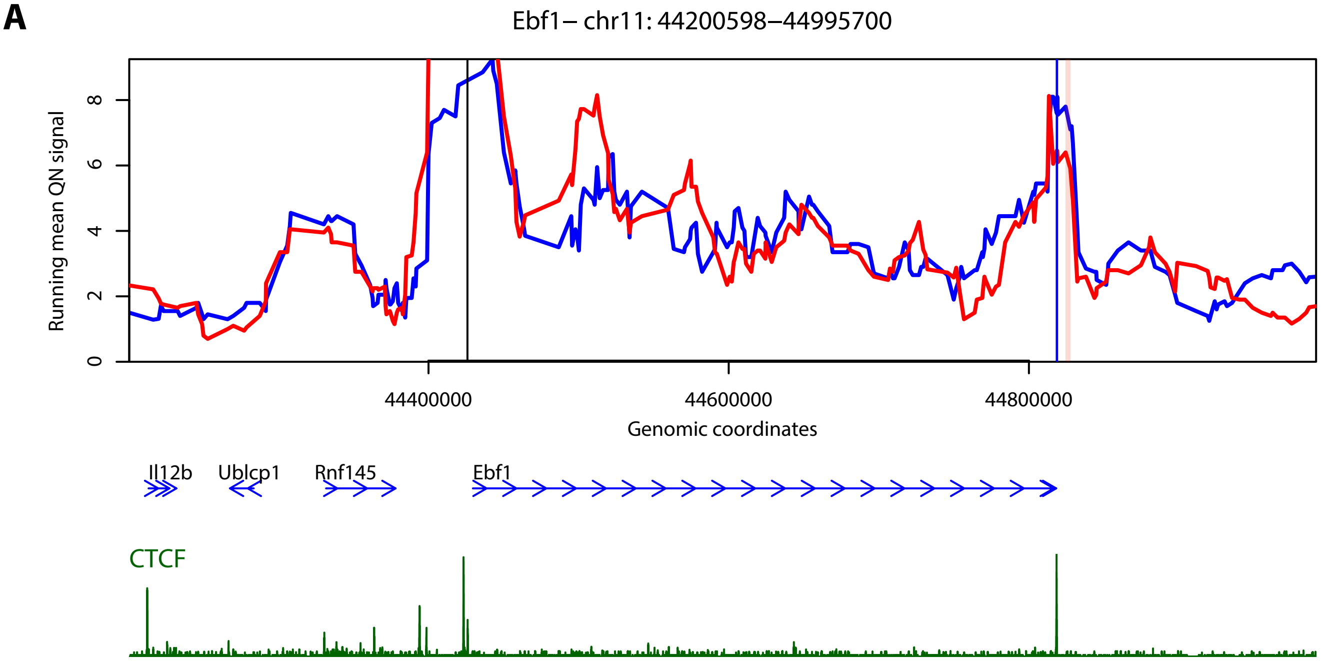
A
Ebf1– chr11: 44200598–44995700
Running mean QN signal
0
2
4
6
8
44400000
44600000
44800000
Genomic coordinates
Il12b
Ublcp1
Rnf145
Ebf1
CTCF

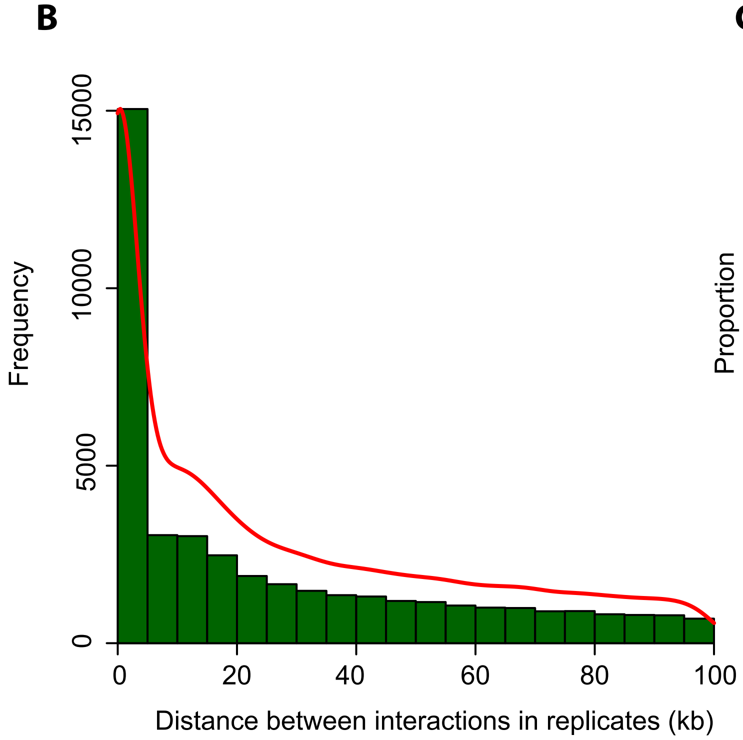
B
Frequency
0
5000
10000
15000
0
20
40
60
80
100
Distance between interactions in replicates (kb)

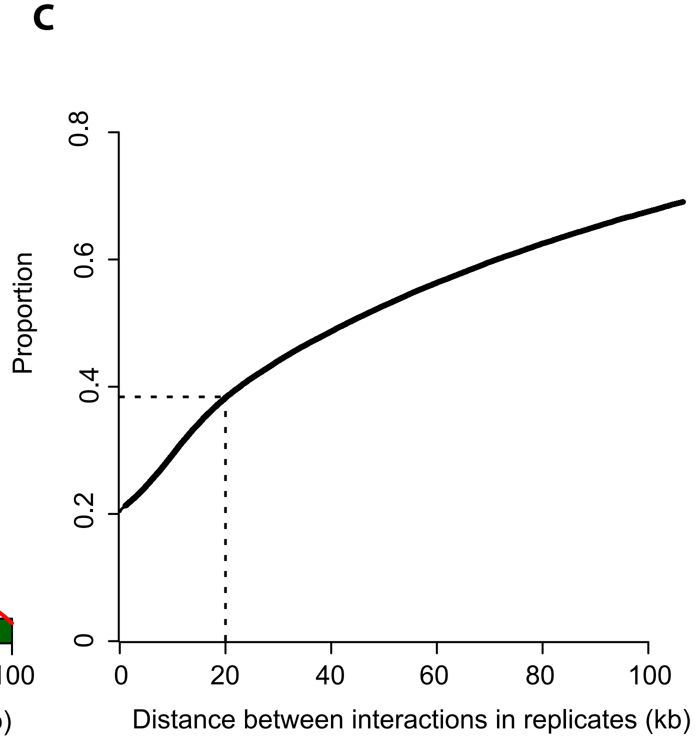
C
Proportion
0
0.2
0.4
0.6
0.8
0
20
40
60
80
100
Distance between interactions in replicates (kb)

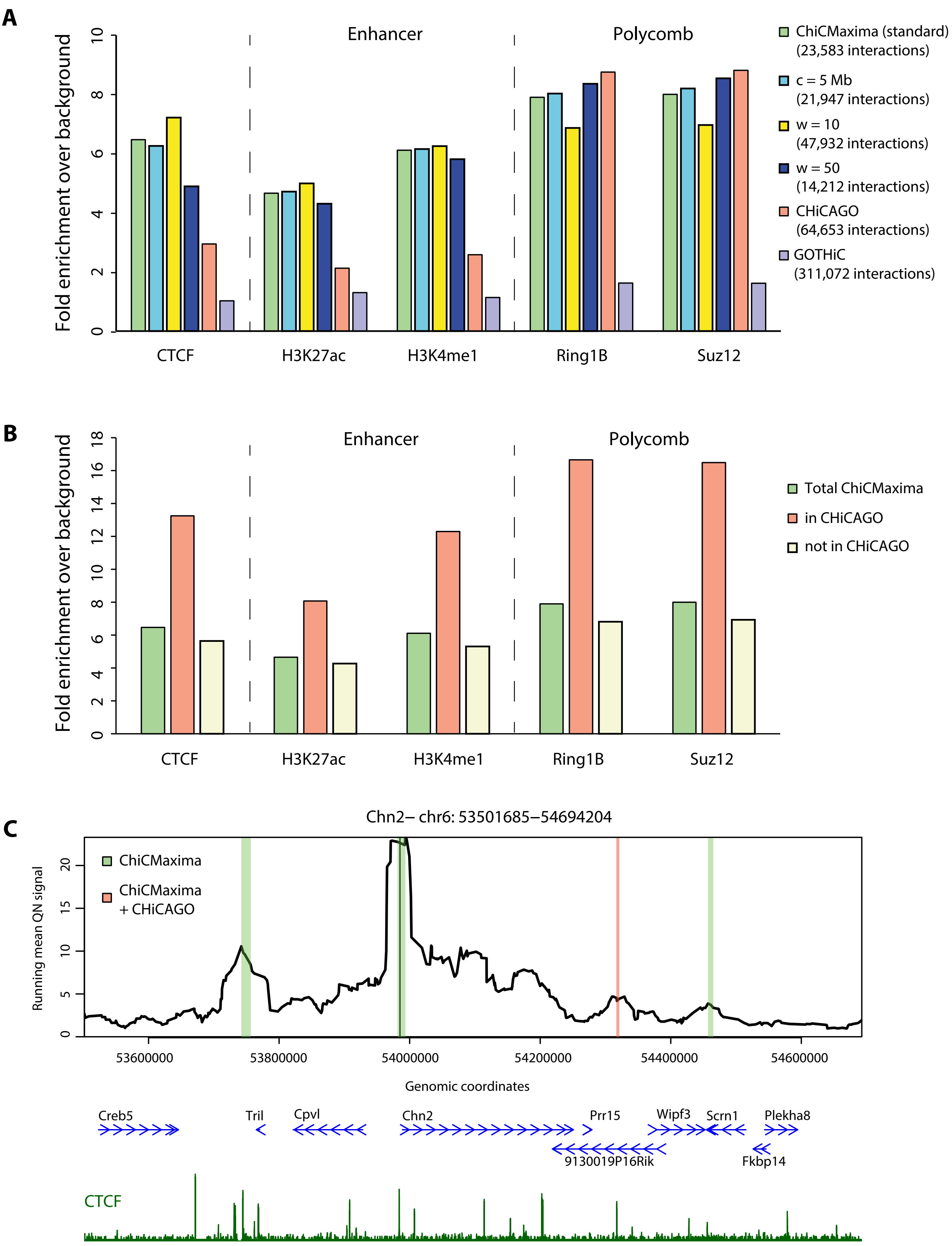
A
Fold enrichment over background
0
2
4
6
8
10
Enhancer
Polycomb
CTCF
H3K27ac
H3K4me1
Ring1B
Suz12
ChiCMaxima (standard) (23,583 interactions)
c = 5 Mb (21,947 interactions)
w = 10 (47,932 interactions)
w = 50 (14,212 interactions)
CHiCAGO (64,653 interactions)
GOTHiC (311,072 interactions)
B
Fold enrichment over background
0
2
4
6
8
10
12
14
16
18
Enhancer
Polycomb
CTCF
H3K27ac
H3K4me1
Ring1B
Suz12
Total ChiCMaxima
in CHiCAGO
not in CHiCAGO
C
Chn2– chr6: 53501685–54694204
Running mean QN signal
0
5
10
15
20
ChiCMaxima
ChiCMaxima + CHiCAGO
53600000
53800000
54000000
54200000
54400000
54600000
Genomic coordinates
Creb5
Tril
Cpvl
Chn2
Prr15
Wipf3
Scrn1
Plekha8
9130019P16Rik
Fkbp14
CTCF

Ben Zouari et al. Fig S5

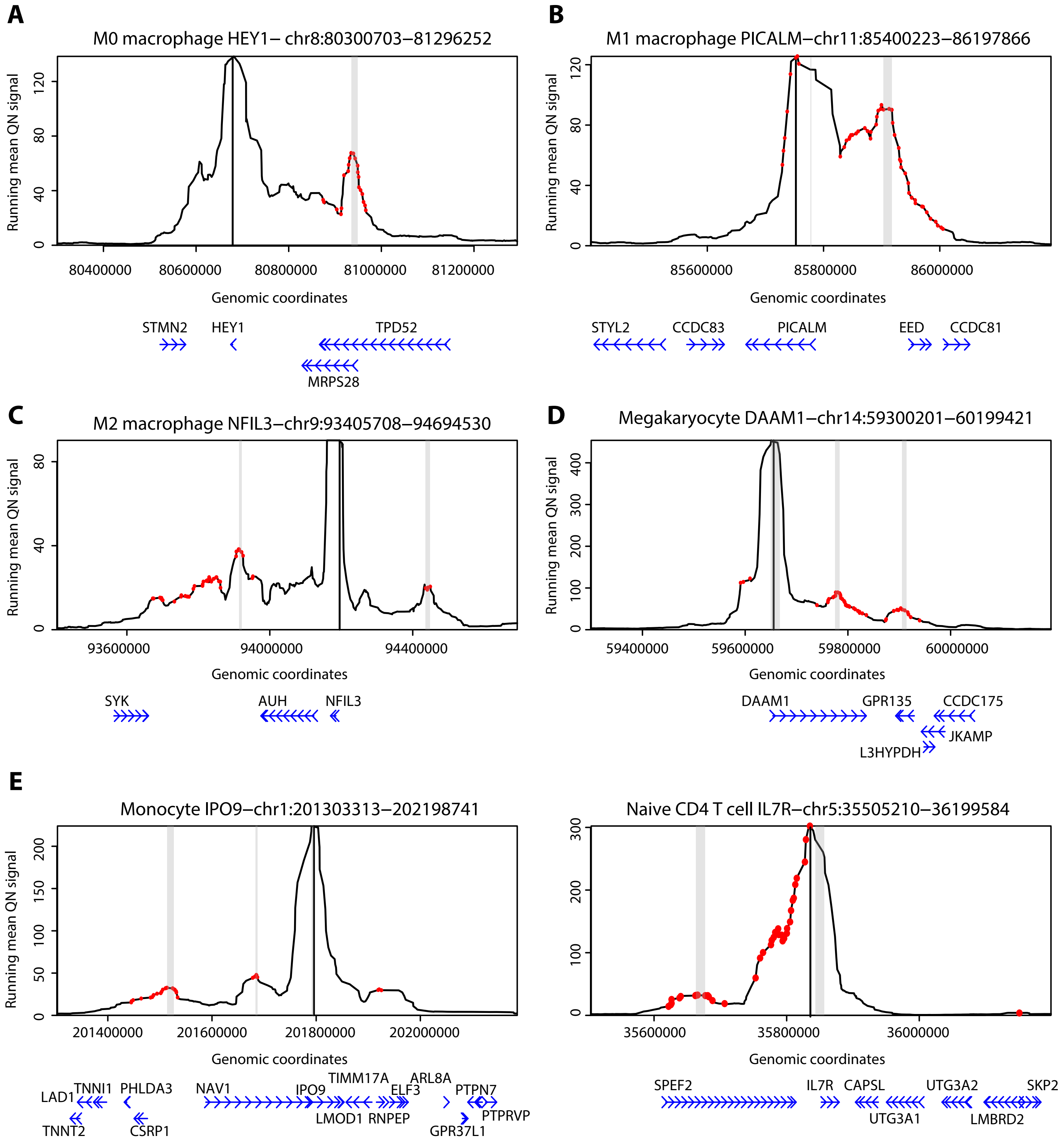

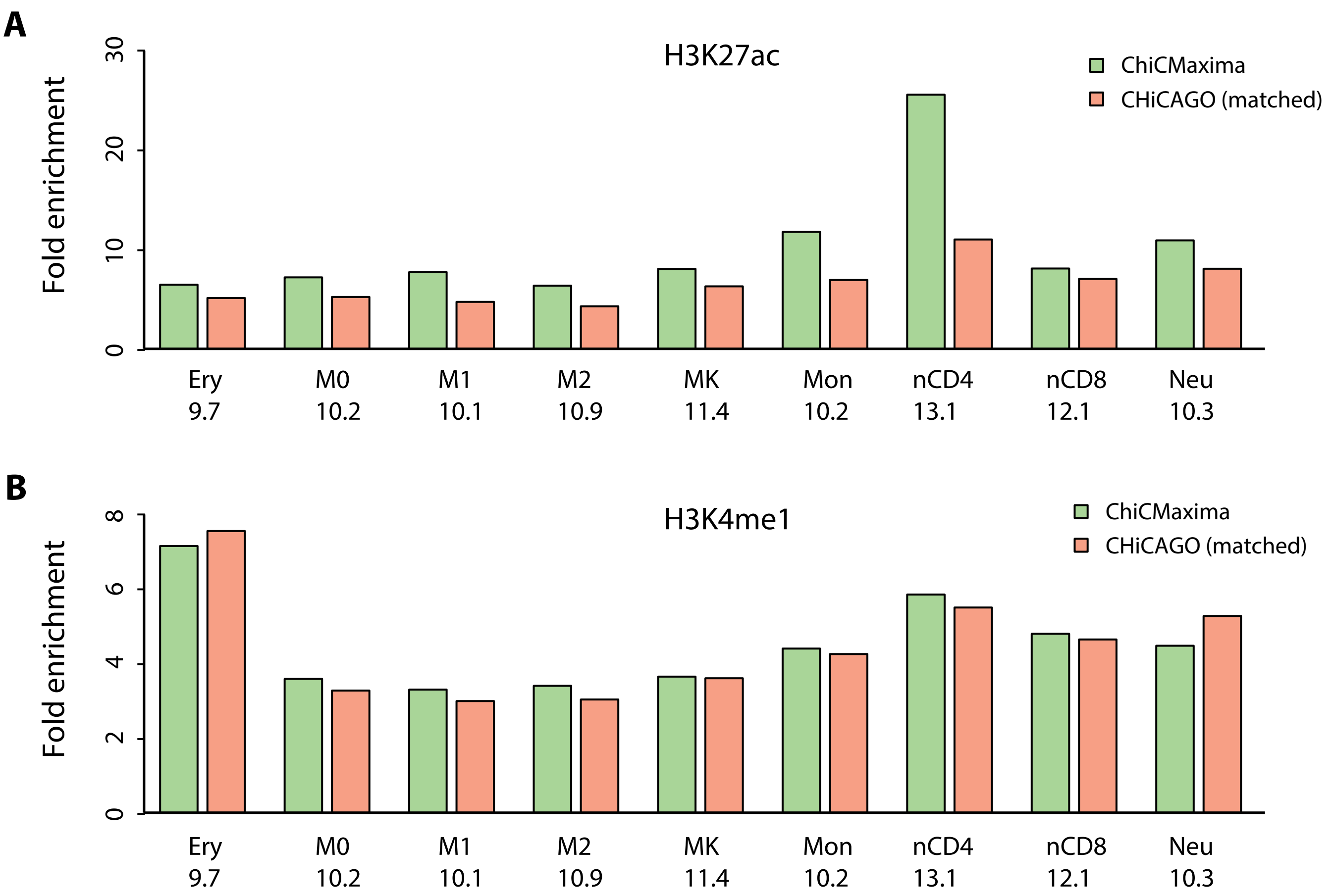
A
H3K27ac
ChiCMaxima
CHiCAGO (matched)
Fold enrichment
0
10
20
30
Ery
9.7
M0
10.2
M1
10.1
M2
10.9
MK
11.4
Mon
10.2
nCD4
13.1
nCD8
12.1
Neu
10.3
B
H3K4me1
ChiCMaxima
CHiCAGO (matched)
Fold enrichment
0
2
4
6
8
Ery
9.7
M0
10.2
M1
10.1
M2
10.9
MK
11.4
Mon
10.2
nCD4
13.1
nCD8
12.1
Neu
10.3

Ben Zouari et al. Fig S7

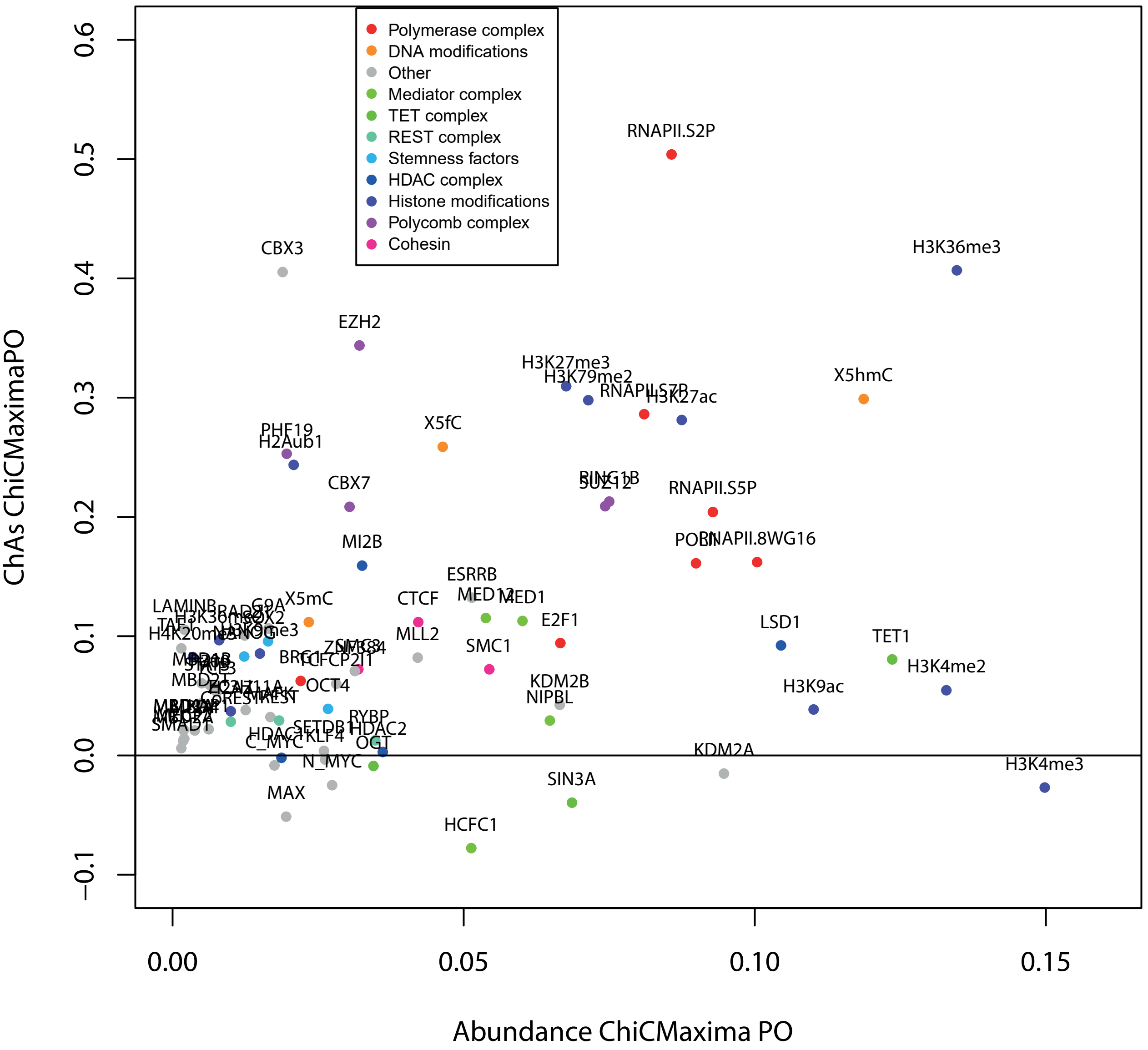

Ben Zouari et al. Fig S8

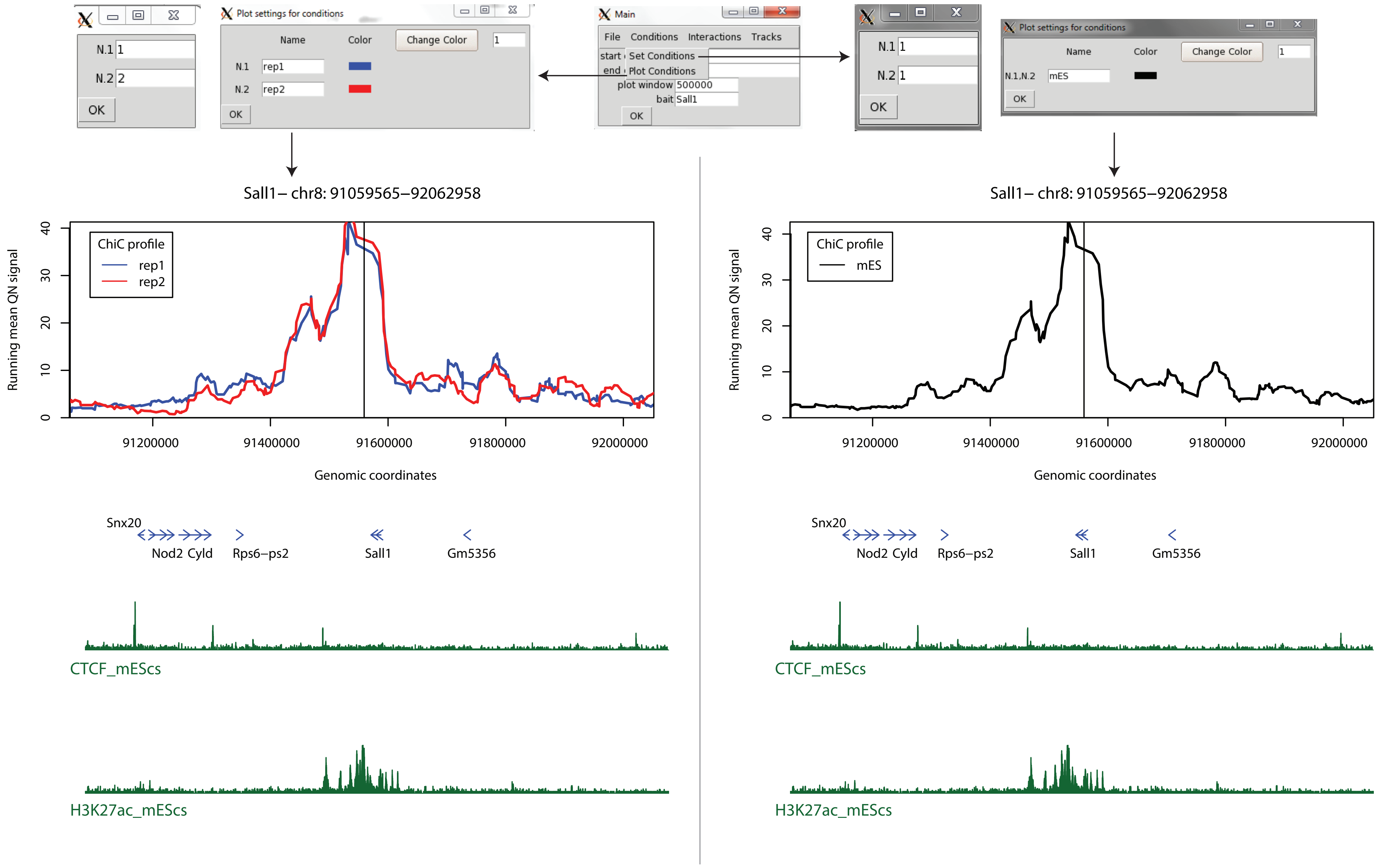

SUPPLEMENTARY FIGURE LEGENDS

Figure S1. Under-sampled CHi-C datasets confound analyses at single restriction fragment level. **a** Venn diagrams of interactions from two biological replicates of mES CHi-C, called separately by CHiCAGO (left) or ChiCMaxima (right). Although at a single-replicate level, CHiCAGO calls about ten-fold more interactions than ChiCMaxima, fewer total interactions are conserved across two biological replicates at the single restriction fragment level. Note that despite giving a 20-fold improvement in reproducibility over CHiCAGO, fewer than 10% of interactions called by ChiCMaxima are conserved across two replicates at the single restriction fragment level. **b** The CHi-C profile centered on the bait *Tet2* promoter is shown for two mES replicates (blue and red); the quantile-normalized raw reads in the top panel, and the quantile-normalized running mean (over ten fragment windows) in the lower panel. Less reproducible signal "spikes" at single fragments in the raw reads are called as interactions by replicate-specific CHiCAGO analyses (red and blue stripes for each replicate, respectively; CHiCAGO score ≥ 5); an interaction is called by ChiCMaxima (gray stripe) over both replicates at a CTCF-rich region, based on consistently higher CHi-C signal over consecutive restriction fragments. The gene positions (blue) and mES ChIP-seq profiles for CTCF and H3K27ac (green) are shown below the CHi-C profiles.

Figure S2. Testing parameters of ChiCMaxima. **a** Boxplot showing distribution of read numbers for interactions called by ChiCMaxima with different settings of *w* within a subset of mES CHi-C data (no extra filters applied). Total numbers of called interactions is given below the boxes. Increasing *w* increases the number of supporting reads within fewer overall called interactions. All *w* settings tested gave interaction calls with overall greater numbers of reads

than the calls made by CHiCAGO on the same dataset. **b** Boxplot showing distribution of interaction distances for the same ChiCMaxima calls as in **a**. Large *w* has a strong bias towards shorter-range interactions. **c** Boxplot showing distribution of read numbers for interactions called by ChiCMaxima on the same dataset as **a**, with *w* = 20, and applying different filters (None – no filter; Geo – filtering for reads greater than geometric mean within 20 kb genomic separation bins; Lin – filtering for reads greater than log-linear fit to same geometric mean distribution; Cub – filtering for cubic fit to log-distance of same geometric mean distribution). **d** Boxplot showing distribution of interaction distances for the same ChiCMaxima calls as in **c**. We note that applying a geometric mean filter to smaller *w* introduces much less bias to shorter-range interactions than applying no filter to large *w*.

Figure S3. CHi-C interaction calling across biological replicates**. a** The CHi-C profile (quantile-normalized running mean over ten fragments) centered on the bait *Ebf1* promoter is shown for two mES replicates (blue and red). A selected interaction with a CTCF site is highlighted, with the exact position of the called ChiCMaxima interaction shown for each replicate (replicate 1 in blue; replicate 2 in red). This interaction is not reproduced across the two replicates at the individual restriction fragment level, but the two calls are within 5650 bp/4 *Hind*III fragments of each other, and centered on the same major peak. The gene positions (blue) and mES ChIP-seq profile for CTCF (green) are shown below the CHi-C profiles. **b** Histogram showing the distributions for the genomic distances between interacting regions called from one replicate and the closest interaction called from the other replicate. **c** Cumulative frequency plot for the same genomic distance distribution as **b**; nearly 40% of the interactions of one replicate are within 20 kb of the interactions of the other replicate.

Figure S4. Epigenomic enrichments from alternative interaction calling methods. **a** Bar chart showing fold enrichment over genomic background for different ChIP-seq peaks within all the promoter-interacting sequences determined by ChiCMaxima with different parameters. Whichever ones are used, the enrichments are consistently higher for enhancer marks than either CHiCAGO- or GOTHiC-called interactions, and comparable to CHiCAGO for Polycomb marks. **b** Bar chart showing fold enrichment over genomic background for different ChIP-seq peaks within all the promoter-interacting sequences determined by ChiCMaxima, compared to the subsets that are or are not conserved with interactions called by CHiCAGO. **c** The CHi-C profile (quantile-normalized running mean over ten fragments) centered on the bait *Chn2* promoter, with interactions called by ChiCMaxima alone denoted with green stripes, and interactions called by both ChiCMaxima and CHiCAGO denoted with orange stripes. Gene position (blue) and the mES CTCF ChIP-seq profile (dark green) are shown below the CHi-C profile. Interactions with additional CTCF sites are called by ChiCMaxima, which are not recapitulated by CHiCAGO.

Figure S5. Improved stringency of ChiCMaxima over CHiCAGO when applied to human primary hematopoietic cell CHi-C data. Several CHi-C profiles are plotted for different cell type/gene promoters, with interactions called by CHiCMaxima denoted as gray stripes, and interactions called by CHiCAGO denoted as red spots. Gene positions (blue) are shown below the profiles. **a** *HEY1* in M0 macrophages; **b** *PICALM* in M1 macrophages; **c** *NFIL3* in M2 macrophages; **d** *DAAM1* in megakaryocytes; **e** *IPO9* in monocytes; **f** *IL7R* in naïve CD4 T cells.

Figure S6. ChiCMaxima is not just a more stringent version of CHiCAGO. Bar charts showing fold enrichment over genomic background for ChIP-seq peaks (**a** H3K27ac; **b** H3K4me1) within all the promoter-interacting sequences determined by ChiCMaxima, compared to the equal number of highest-scoring CHiCAGO-called interactions, computed across nine human primary hematopoietic cell CHi-C datasets. The numbers below the bar chart denote the threshold CHiCAGO score applied to obtain the required number of interactions. ChiCMaxima nearly always outperforms the matched CHiCAGO calls.

Figure S7. Scatter plot of chromatin assortativity against relative feature abundance for different chromatin features within the ChiCMaxima-called interaction network derived from the mES CHi-C dataset.

Figure S8. Flexibility in handling replicates in ChiCBrowser. The CHi-C profiles plotted from two mES CHi-C biological replicates (called “N.1” and “N.2” in the original input file) by ChiCBrowser for a 1 Mb window centered on the bait *Sall1* promoter. Left: The replicates are plotted side by side by giving N.1 and N.2 different levels (1 and 2, respectively) in the *Set Conditions* menu, and renaming them “rep1” and “rep2” in the *Plot Conditions* menu. Right: The mean of the two replicates is plotted by giving N.1 and N.2 the same level (1) in the *Set Conditions* menu, and renaming the combined plot “mES” in the *Plot Conditions* menu.

Table S2: Overview of CHi-C interactions called by ChiCMaxima with different parameters.

	**ChiCMaxima (standard)**	***c* = 5 Mb**	***w* = 10**	***w* = 50**
**Number of called interactions**	23583	21947	47932	14212
**Mean number of called interactions per bait**	1.4	1.37	2.5	1.05

Table S3: Overview of putative mES enhancers found within CHi-C interactions called by ChiCMaxima with varying parameters.

	**Putative mES enhancers in called interaction set**	**Total called interactions / interactions with putative enhancers**
**ChiCMaxima (standard)**	16.8% (3235)	7.3
***c* = 5 Mb**	16.1% (3092)	7.1
***w* = 10**	33.0% (6341)	7.6
w = 50	9.7% (1868)	7.6
