# Supplementary material for: ChiCMaxima: a robust and simple pipeline for detection and visualization of chromatin looping in Capture Hi-C
Source: Genome Biol. 2019 May 22;20:102. doi: 10.1186/s13059-019-1706-3 (PMC6532271; doi:10.1186/s13059-019-1706-3)
Supplement: Supplementary file 2 — ChiCMaxima and ChiCBrowser user guide. (PDF 355 kb) [file 13059_2019_1706_MOESM2_ESM.pdf]

ChiCMaxima: a robust and simple pipeline for detection and visualization of chromatin looping in Capture Hi-C

Additional File 2: ChiCMaxima and ChiCBrowser User Guide

Yousra Ben Zouari, Anne Molitor, Natalia Sikorska, Vera Pancaldi and Tom Sexton

Contents

1. System requirements and test data

2. Input format

3. Calling interactions on single experiments - ChiCMaxima_Caller

4. Exploring distance thresholds for biological replicates - ChiCMaxima_RepAnalysis

5. Merging biological replicates - ChiCMaxima_MergeRep2 and ChiCMaxima_MergeRepMany

6. Collating experiments into browser input file - ChiCMaxima_Collate

7. Browsing CHi-C data - ChiCBrowser

1. System requirements and test data

All parts of the ChiCMaxima suite and the ChiCBrowser is run on R, version >=3.2. These packages (and their dependencies) are additionally required, and can be found in Bioconductor or CRAN:

- GenomicRanges
- MASS
- data.table
- zoo
- psych
- tcltk2
- tkrplot
- limma
- caTools
- rtracklayer

For use of the *utils* scripts (see below), perl is required, and one script converting BAM files to ChiCMaxima inputs (see section 2) also requires installation of bedtools [1] and for a route to it to be present in $PATH.

The following files have been included in the folder *testdata* for the user to trial the scripts from ChiCMaxima and ChiCBrowser:

- mESrep1_chr15.ibed and mESrep2_chr15.ibed. Two replicate CHi-C mES datasets (from [2]), in the appropriate ibed input format (see section 2) for ChiCMaxima, restricted to chromosome 15.
- otherinteractions_chr15.ibed. Interactions called by ChiCMaxima_Caller for an unrelated cell type, restricted to chromosome 15 (unpublished results from our group). The sole purpose of these data here is to provide a third interaction list for demonstrating the tool ChiCMaxima_MergeRepMany.
- collate_key.txt. A table setting the parameters for collating the two replicate CHi-C datasets into one for input into ChiCBrowser (see section 6).
- mEScollated.ibed. The collated biological replicates' CHi-C data (output of section 6), ready for input in ChiCBrowser.
- mm9_genes_chr15.txt. The modified bed file giving the mm9 gene annotations, restricted to chromosome 15, for ChiCBrowser (see section 7).
- CTCF_mEScs.bw and H3K27ac_mEScs.bw. Two mES ChIP-seq profiles in bigWig format for ChiCBrowser (see section 7).

Additionally, files can be found in the folder *utils* which help for converting the outputs of Hi-C analysis pipelines into the appropriate input format for ChiCMaxima (see section 2):

- mm9_HindIII.rmap and mm9_HindIII.baitmap. These files give the chromosomal coordinates for all mouse HindIII fragments (mm9 assembly) and those which contain capture oligonucleotides within the promoter capture design described in [2], respectively.
- bam2ibed.pl and align2ibed.pl scripts, which use the above files to convert Hi-C analysis outputs (BAM files from HiCUP [3], or generic tabular format, respectively) into the ChiCMaxima input format.

2. Input format

As for CHiCAGO [4], ChiCMaxima deals with “interaction BED” or ibed inputs. These are 11-column headed tables:

1. **ID_Bait**: Unique integer ID for the restriction fragment containing the bait.
2. **chr_Bait**: Chromosome containing the bait (in the format *chr1*, *chr2*, …, *chrX*).
3. **start_Bait**, and
4. **end_Bait**: the coordinates of the bait restriction fragment (start < end).
5. **Bait_name**: A name given to the bait (e.g. the gene name for promoter CHi-C). This is used as the search term in ChiCBrowser.
6. **ID_OE**,
7. **chr_OE**,
8. **start_OE**, and
9. **end_OE**: as for columns 1-4, giving the unique integer ID and chromosomal coordinates of the non-bait (other end/OE) fragment.
10. **OE_name**: Should be “.” (i.e. no bait identifier), as bait-to-bait interactions are not supported.
11. **N**: number of sequencing reads for this specific bait-to-non-bait interaction.

As for CHiCAGO, conversion to the ibed format is facilitated by two lookup tables: one between restriction fragment genomic coordinates (**chr, start, end**) and the fragment ID (**ID**), regardless of whether the fragment is a bait (**Bait**) or not (**OE**). The second lookup table links the bait information to its identifier (**Bait_name**). The first lookup table for the worked example is provided in the *utils* folder as mm9_HindIII.rmap: 4-column, non-headed table (Chromosome, in format *chr*1, *chr2*, …, *chrX*; Start; End; ID). The second lookup table for the worked example is provided in the *utils* folder as mm9_HindIII.baitmap: 5-column, non-headed table (Chromosome, in format *chr*1, *chr2*, …, *chrX*; Start; End; ID, which must correspond to the same fragment in the rmap file; Identifier, in this case, the gene name).

All Hi-C analyses, like HiC-Pro [5], generate, at least in a temporary file, a table of paired alignments, whereby the genomic coordinates of one read is associated with the genomic coordinates of its mated pair. The script from *utils*, align2ibed.pl can flexibly convert any table with this minimum information into the ibed format for ChiCMaxima.

Usage perl align2ibed.pl [BEDPE FILE] [BAITMAP FILE] [RMAP FILE] [OUTPUT IBED FILE] [FIRST READ CHR COLUMN] [FIRST READ COORDINATE COLUMN] [SECOND READ CHR COLUMN] [SECOND READ COORDINATE COLUMN].

The input bedpe (bed paired end) file is read in, and the chromosome and coordinate for the two paired reads are extracted from their appropriate columns (given by the user in the last four arguments; columns start at 1, not zero). These are then converted to ibed format, using the bait and restriction map information provided by the baitmap and rmap files. Only intrachromosomal interactions where one read contains a bait and the other does not are kept.

Note that this script is very generic, and not intended to replace existing Hi-C processing pipelines. In particular, known technical artefacts of both Hi-C and CHi-C, such as PCR duplicates or self-ligation events, are not filtered out.

The HiCUP pipeline [3] outputs the analysis in a BAM format. The script bam2ibed.pl uses bedtools [1] to convert the BAM file to a bedpe format, which is then converted to the ibed format by internal calling of align2ibed.pl. Bedtools must be installed and have a route to it in $PATH.

Usage perl bam2ibed.pl [BAM FILE] [BAITMAP FILE] [RMAP FILE] [OUTPUT IBED FILE].

The output file must be given with a ".ibed" suffix; the prefix preceding this is also used to name the intermediate file *prefix*.bedpe.

3. ChiCMaxima_Caller

Brief: Calls CHi-C interactions from single datasets, based on local maximum computation and approximate prediction of cis-decay.

Usage (from folder containing scripts)**:** Rscript ChiCMaxima_Caller.r -i/--input [INPUT IBED FILE] -o/--output [OUTPUT PREFIX] -w/--window_size [LOCAL MAXIMUM CALLING WINDOW] -s/--loess_span [LOESS SPAN] -c/--cis_window [GENOMIC SEPARATION THRESHOLD] -b/--binwidth [DISTANCE SEPARATION BIN]

Arguments:

- --input. 11-column ibed input file (headed or not; see section 2 for format).
- --output. Folder and prefix for output files (called interactions, and list of non-assessed baits).
- --window_size. Number of covered restriction fragments within which the local maximum is called in sliding windows. Default: 50
- --loess_span. The loess span parameter for smoothing the virtual 4C profile. Default: 0.05
- --cis_window. Interactions are assessed within this many bp of the bait start coordinate. Default: 1500000
- --binwidth. The widths, in bp, of the fixed bins used to assess the geometric means for filtering.

What is done:

The input ibed file is read in and checked for the appropriate format. Warning messages are given if bait-to-bait or interchromosomal interactions are found. For each bait individually, a sub-table of the ibed is extracted for all interactions within *cis_window* of the bait start coordinate. Tables with fewer than (2**window_size* + 1) covered fragments cannot be assessed for local maxima, and the names of these poorly covered baits are recorded. If there is sufficient coverage, the profile of N versus genomic coordinate is smoothed by loess smoothing (with a span of *loess_span*). Local maxima within sliding windows of size *window_size* are computed within this profile. If local maxima are found, the local profile is split into genomic separation bins of width *binwidth* (ranging from 0 to *binwidth*, to (*cis_window-binwidth* to *cis_window*), and the geometric mean of the sequence counts falling within each bin is computed to set a threshold for filtering. The called local maxima are filtered to those that have an interaction score higher than the geometric mean for its corresponding genomic separation. The numbers of called interactions and poorly covered baits are written to the standard output.

Outputs:

- *output*_interactions.ibed. The called interactions, in the same ibed format as the input.

- *output*_poorlycovered.txt. A list of the baits with insufficient coverage for local maximum assessment. This and their numbers can be used to inform the user of which -*w* parameter to use.

Example:

Rscript ChiCMaxima_Caller.r -i testdata/mESrep1_chr15.ibed -o testdata/mESrep1

Returns 2613 interactions, with 33 poorly covered baits.

Rscript ChiCMaxima_Caller.r -i testdata/mESrep2_chr15.ibed -o testdata/mESrep2

Returns 1604 interactions, with 65 poorly covered baits.

4. ChiCMaxima_RepAnalysis

Brief: Computes the distributions of closest distances between called interactions within two outputs of CHiCMaxima_Caller. The goal is to help the user choose an appropriate -*d* setting for ChiCMaxima_MergeRep2/ChiCMaxima_MergeRepMany (see section 5).

Usage (from folder containing scripts): Rscript ChiCMaxima_RepAnalysis.r -a/--file1 [INTERACTIONS FILE 1] -b/--file2 [INTERACTIONS FILE 2] -o/--output [OUTPUT PREFIX]

Arguments:

- --file1, and
- --file2. The output interaction files from ChiCMaxima_Caller, in the headed 11-column ibed format.
- --output. Folder and prefix for output files (histogram, cumulative frequency plot, quantile table).

What is done:

Both interaction files are read in, and bait that have called interactions in both are used for further bait-by-bait analysis. Sub-tables of interactions with bait-specific interactions from each replicate are taken. For the replicate with the fewest numbers of interactions, the genomic distance to the closest other replicate interaction is recorded. These closest distance distributions are output as a histogram, as a cumulative frequency plot, and as a table giving the genomic distance for every fifth percentile (i.e. 0, 5, 10, …, 90, 95, 100).

Outputs:

- *output*_hist.png. The histogram for the closest distance distribution.
- *output*_ecdf.png. The cumulative frequency plot for closest distances.
- *output*_percentiles.txt. The table giving the genomic distance for every fifth percentile.

Example:

Rscript ChiCMaxima_RepAnalysis.r -a testdata/mESrep1_interactions.ibed -b testdata/mESrep2_interactions.ibed -o testdata/mEScombined.

Returns the histogram, cumulative frequency plot and percentiles table (25th percentile is 0; median is 19743 bp; 75th percentile is 81189 bp).

5a. ChiCMaxima_MergeRep2

Brief: Filters the called interactions from two biological replicates to only include those within a threshold genomic distance.

Usage (from folder containing scripts): Rscript ChiCMaxima_MergeRep2.r -a/--onepeak [INTERACTIONS FILE 1] -b/--twopeak [INTERACTIONS FILE 2] -d/--repdist [THRESHOLD DISTANCE] -o/--output [OUTPUT IBED FILE]

Arguments:

- --onepeak, and
- --twopeak. The output interaction files from ChiCMaxima_Caller, in the headed 11-column ibed format.
- --repdist. The maximum genomic distance between the closest interactions within the two replicates for the interaction to be maintained. Default: 0
- --output. The file name for the output ibed file of the merged, filtered interactions list.

What is done:

Both interaction files are read in, checked for the appropriate 11-column ibed format, and that they share baits in common. For each bait in common, sub-tables of interactions with bait-specific interactions from each replicate are taken. For the replicate with the fewest numbers of interactions, the closest other replicate interaction is found, and this interaction is kept if the distance between the two replicates is less than or equal to *repdist*. These interactions are stored as a merged ibed format: ID_Bait, chr_Bait, start_Bait, end_Bait, Bait_name, chr_OE, start_OE, end_OE, OE_name, N.1, N.2. Due to the flexible window, an ID_OE is no longer applicable and the coordinates of start_OE and end_OE give the limits of the merged interactions. The N values for each replicate are maintained. The total number of merged interactions is written to the standard output.

Outputs:

- *output*. The merged ibed file (see above for column headings).

Example:

Rscript ChiCMaxima_MergeRep2.r -a testdata/mESrep1_interactions.ibed -b testdata/mESrep2_interactions.ibed -d 20000 -o testdata/mEScombined_interactions.ibed

Returns 775 merged interactions.

5b. ChiCMaxima_MergeRepMany

Brief: Filters the called interactions from three or more biological replicates to only include those within a threshold distance.

Usage (from folder containing scripts): Rscript ChiCMaxima_MergeRepMany.r [THRESHOLD DISTANCE] [MAX_OE_WIDTH] [OUTPUT IBED FILE] [INTERACTIONS FILE 1] [INTERACTIONS FILE 2] [INTERACTIONS FILE 3] … [INTERACTIONS FILE *n*]

Arguments (note that these do not come with a "-" or "--" prefix):

- THRESHOLD DISTANCE. As --*repdist* in ChiCMaxima_MergeRep2. There is no default setting.

- MAX_OE_WIDTH. Maximum allowed size of final OE called region, to avoid "daisychaining" (see below)
- OUTPUT IBED FILE. As --*output* in ChiCMaxima_MergeRep2.
- INTERACTIONS FILES. A list of three or more interaction ibed files for merging.

What is done:

All interaction replicate files are read and checked for 11-column ibed format; the baits conserved in all replicates are used for bait-by-bait analysis. The sub-tables of the bait-specific interactions are taken, and the replicate with the fewest interactions is used as a *query* against the other replicate *subjects*. For each subject set, the closest interaction to each query interaction is found, and filtered to only keep those that are within *repdist* distance. Each time, the start_OE and end_OE coordinates are modified to account for the merged replicates. The interactions conserved across all replicates are kept. Due to the possibility of "daisy-chaining", whereby one replicate is within *repwindow* of a second replicate, and the second replicate is within *repwindow* of the third, but in the same orientation (so the first and third are actually quite distal), very large OE coordinates could be called with multiple biological replicates. An extra filter is therefore applied, setting a maximal size of the final called OE region, MAX_OE_WIDTH (i.e. end_OE - start_OE <= MAX_OE_WIDTH). The number of merged and kept interactions are recorded, and they are output to *output*: ID_Bait, chr_Bait, start_Bait, end_Bait, Bait_name, chr_OE, start_OE, end_OE, OE_name, N.1 … N.x, N.mean. The OE coordinates reflect the merged interaction, and as well as all replicate N values being output, the mean value across the replicates is also recorded.

Outputs:

- *output*. The merged ibed file (see above for column headings).

Example:

Rscript ChiCMaxima_MergeRepMany.r 20000 30000 testdata/3combinedinteractions.ibed testdata/mESrep1_interactions.ibed testdata/mESrep2_interactions.ibed testdata/otherinteractions_chr15.ibed

Returns 563 merged interactions.

6. ChiCMaxima_Collate

Brief: Reads in the input ibed files from different CHi-C experiments and collates them into one large ibed file with separate N columns for each dataset, suitable as input for ChiCBrowser.

Usage (from folder containing scripts): Rscript ChiCMaxima_Collate.r -k/--key [COLLATE KEY] -o/--output [OUTPUT IBED FILE]

Arguments:

- --key. File name of a user-provided table which gives the names of all the input ibed files to be collated, along with their unique identifiers for the experiment-specific N column. As shown in testdata/collate_key.txt, this is a non-headed 2-column table: IBED FILE, IDENTIFIER.
- --output. File name for the output collated ibed file.

What is done:

Each ibed file as defined in the *key* is read in (headed or not) and checked for the 11-column format. The *N* column is renamed to the appropriate identifier - *N.[IDENTIFIER]*. These multiple ibed files are then merged into a single ibed, with the first ten columns, followed by each N.IDENTIFIER column. All interactions are maintained, so some N.IDENTIFIER entries will contain zeros, for specific bait-to-non-bait interactions which have reads in some of the experiments but not in others.

Outputs:

- *output.* Headed columns: ID_Bait, chr_Bait, start_Bait, end_Bait, Bait_name, ID_OE, chr_OE, start_OE, end_OE, OE_name, N.[IDENTIFIER 1], N.[IDENTIFIER 2], etc.

Example

Rscript ChiCMaxima_Collate.r -k testdata/collate_key.txt -o testdata/testcollate.ibed

Outputs an ibed file, with N columns N.1 and N.2, identical to that already provided: testdata/mEScollated.ibed

7. ChiCBrowser.

To run the browser, go to the folder containing the scripts and run the R environment. Then use the command:

source("ChiCBrowser.r")

This will open two graphical windows, one where the profile will be output:

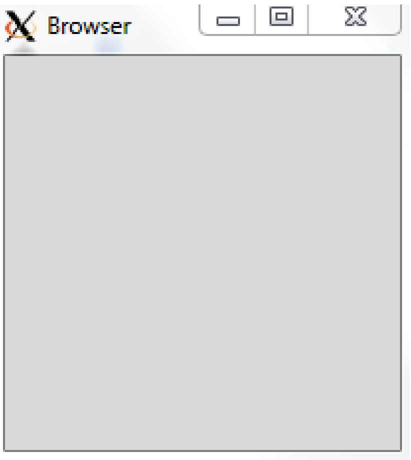

And a control panel:

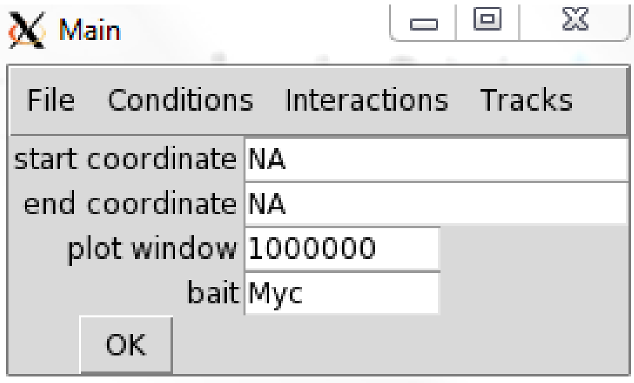

The control panel contains several pull-down menus, explained below.

7a. File

This menu has three commands.

1. New. Clicking this opens a dialog box to select a collated ibed file to load into the memory. Going to the testdata folder and selecting mEScollated.ibed will load the test CHi-C data subset. This file must contain the first ten columns of an ibed format, followed by a uniquely headed column for each experiment's "N" or reads.

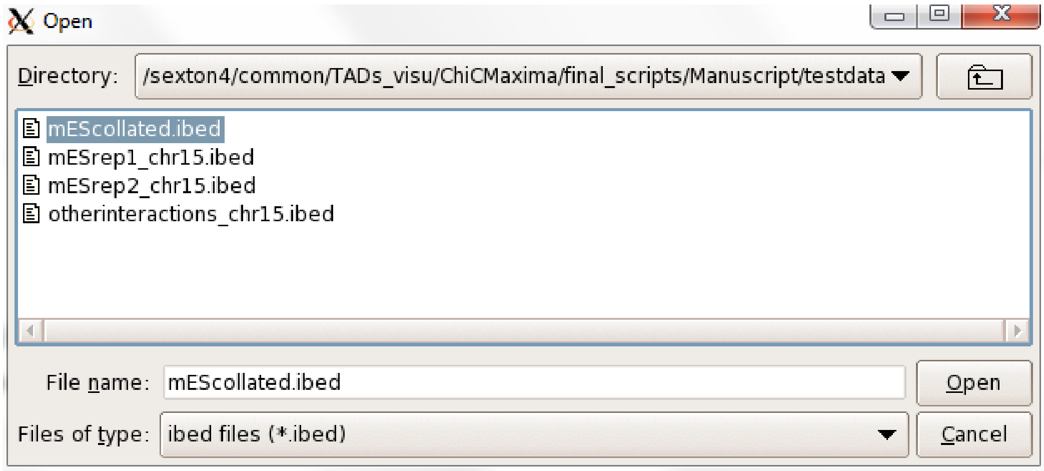

Once this is loaded, a new dialog box opens for the user to select the *Conditions* settings to use (see also section 7b).

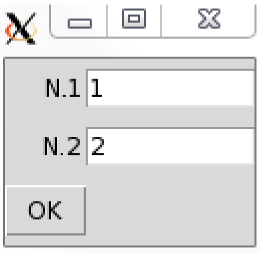

In this case, there are two N columns, N.1 and N.2, giving the sequence reads for two biological replicates of the same condition (mES CHi-C). By selecting N.1 as the level "1" and N.2 as the level "2", the user specifies that they want both replicates to be plotted separately. If they were both given the same level, "1", then one plot would be made of the mean of these two profiles (see Additional File 1: Figure S8). Giving any of the N columns a level of "0" sets it to not be plotted. All other levels must be positive, consecutive integers (1, 2, 3, etc.).

2. Save Image. When a profile screenshot is ready, clicking this option opens up a dialog box for the profile to be saved as an electronic postscript (.eps) file.

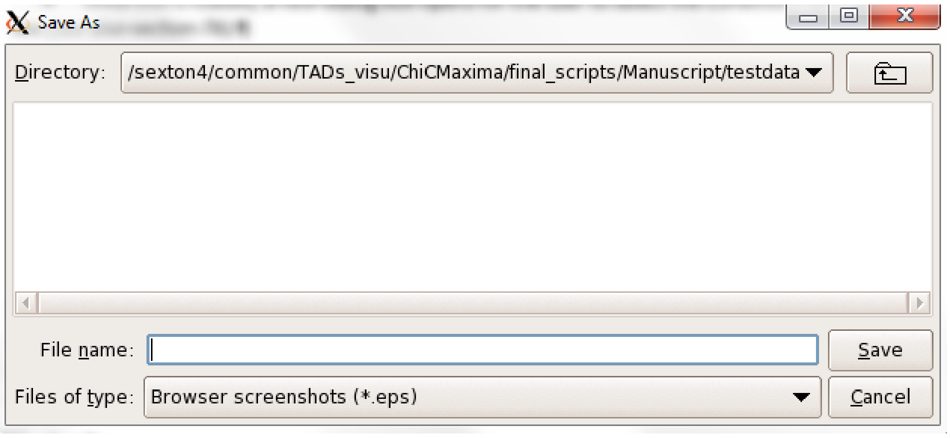

3. Quit. Closes the graphical windows.

7b. Conditions

This menu has two options conferring flexibility on the way to handle different conditions/biological replicates.

1. Set Conditions. Opens the same dialog box as when the ibed file has just been opened (File -> New). This avoids the need to re-load large files in order to change the conditions settings.
2. Plot Conditions. Opens a dialog box like this:

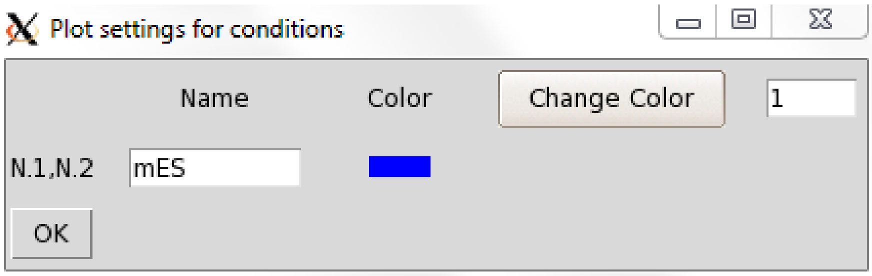

For each condition level (in this case, there is only one, combining the N.1 and N.2 profiles), the leftmost text gives a comma-delimited list of the N columns that are used within. A text box allows the user to give these levels different names for the plot. The color of the plot can also be modified. The user selects the level to modify in the right text panel (from 1 to the number of different levels shown; here, only "1" is available), then clicks the "Change Color" button to open the following dialog box, which has numerous means of controlling the color:

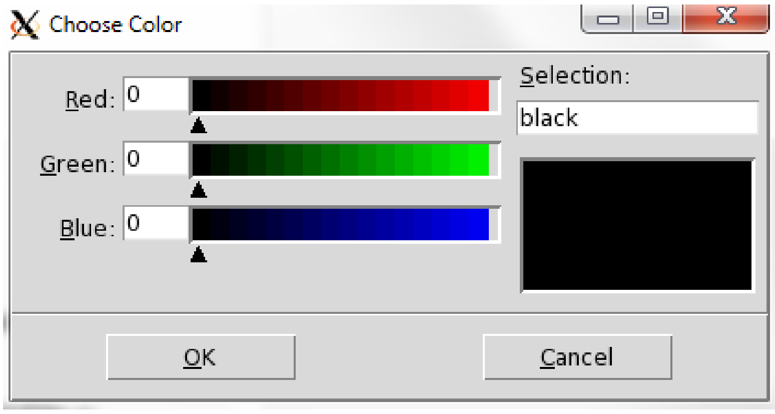

Note, that the exact nature of this panel (and the different options) can vary between Windows and Unix systems (here is shown a Unix version).

7c. Interactions

This menu has two options for handling the highlighting of called interactions (or other, user-defined subsets of the CHi-C dataset):

1. Load Interactions. Opens a dialog box to open one or more ibed files. The required file format is quite flexible, but must be a headed table, with the following columns: Bait_name, start_OE, end_OE. In this example, one can load the merged mES replicate interactions file and the interactions file including the third, unrelated CHi-C dataset:

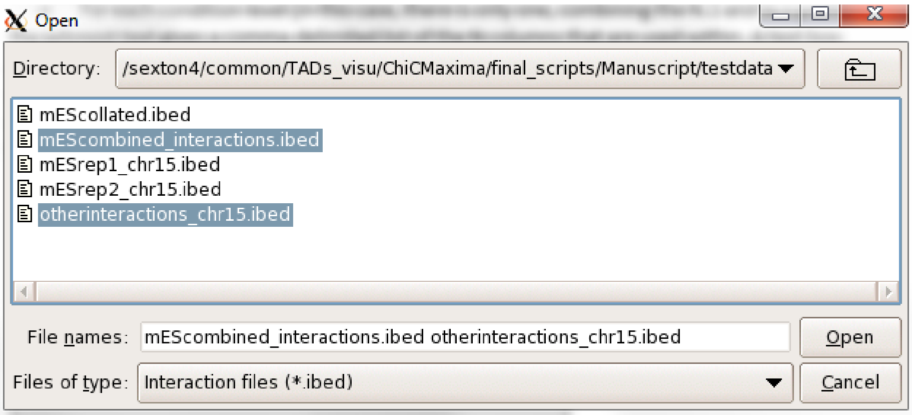

2. Manage Interaction Plots. Similar to the *Conditions*, opens a dialog box, where the different interaction sets can be renamed, their colors can be changed, and whether or not they are included in the plot can be toggled. In this example, the names have been changed to “mES” and “3sets”, of which only “mES” is to be plotted (in blue).

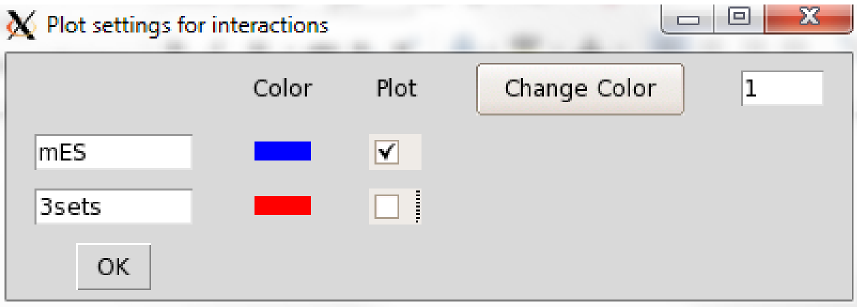

7d. Tracks

This menu has three options for loading and handling other annotations/epigenomic profiles in the plot:

1. Load Genes. Opens a dialog box to load a gene annotation file. In the testdata folder, one can load mm9_genes_chr15.txt. The required format is a headed, tab-delimited text file, with the following columns: Name, Chr, Start, End, Strand. The Chr is given in the format *chr1, chr2, …, chrX*, and the strand must be “+” or “-“. Only one genes file can be loaded.

2. Load Tracks. Opens a dialog box to load one or more epigenomic dataset files. ChiCBrowser is optimized for bigWig or bedGraph files, but any format supported by the *import* function of the rtracklayer package should work. In this case, the user can load the CTCF and H3K27ac mES tracks from the testdata folder.

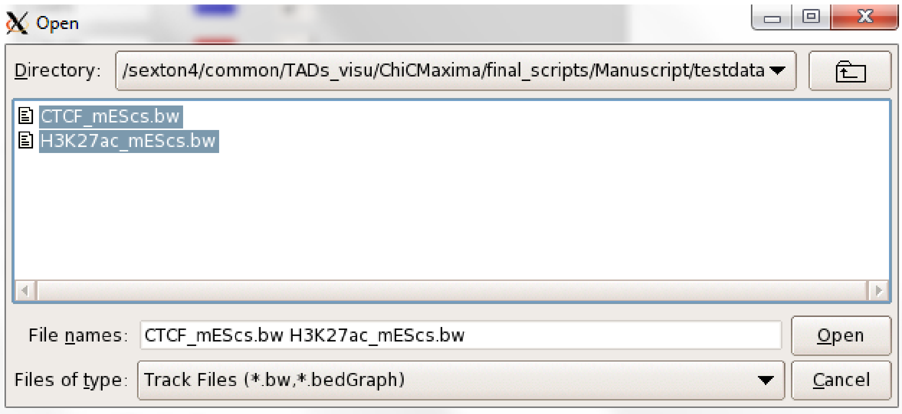

Once these are loaded into the memory, a dialog box is opened, which is identical to that opened by the pull-down menu:

3. Manage Tracks.

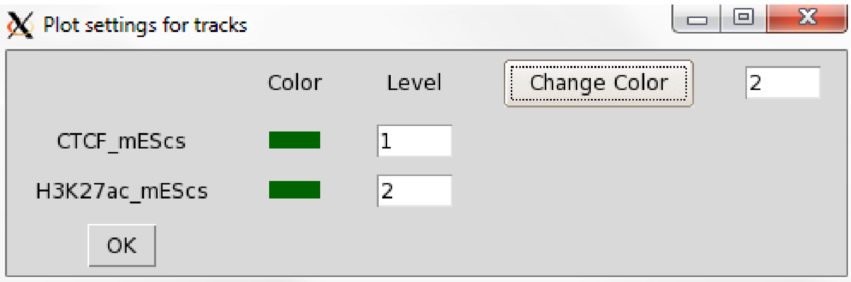

The color and plotting level for each individual track can be altered by the user. As before, the plotting levels can be 0 (not plotted), or consecutive, positive integers. When tracks have the same level, their plots are auto-scaled to the maximum value of all of the included datasets. This feature allows fairer comparison for the same epigenetic mark across different conditions/tissue types; in this case, it is not meaningful to treat two completely different marks on the same scale, so they are given different levels.

7e. The control panel.

Once all the conditions have been set, the control panel has options to choose:

- Start coordinate and end coordinate. Defines the exact chromosomal coordinates within which the profile is plotted.
- Plot window. As an alternative, the user can specify that the plot be a specific size up- and downstream of the bait coordinate. If start and end coordinates have been set, they override the plot window setting.
- Bait. The specific bait to be used in the profile. This must match exactly the name of an entry in the Bait_name column of the loaded ibed file (case-sensitive).

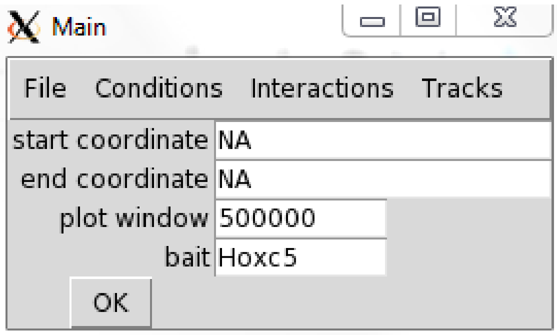

The plot is then generated by clicking the OK button:

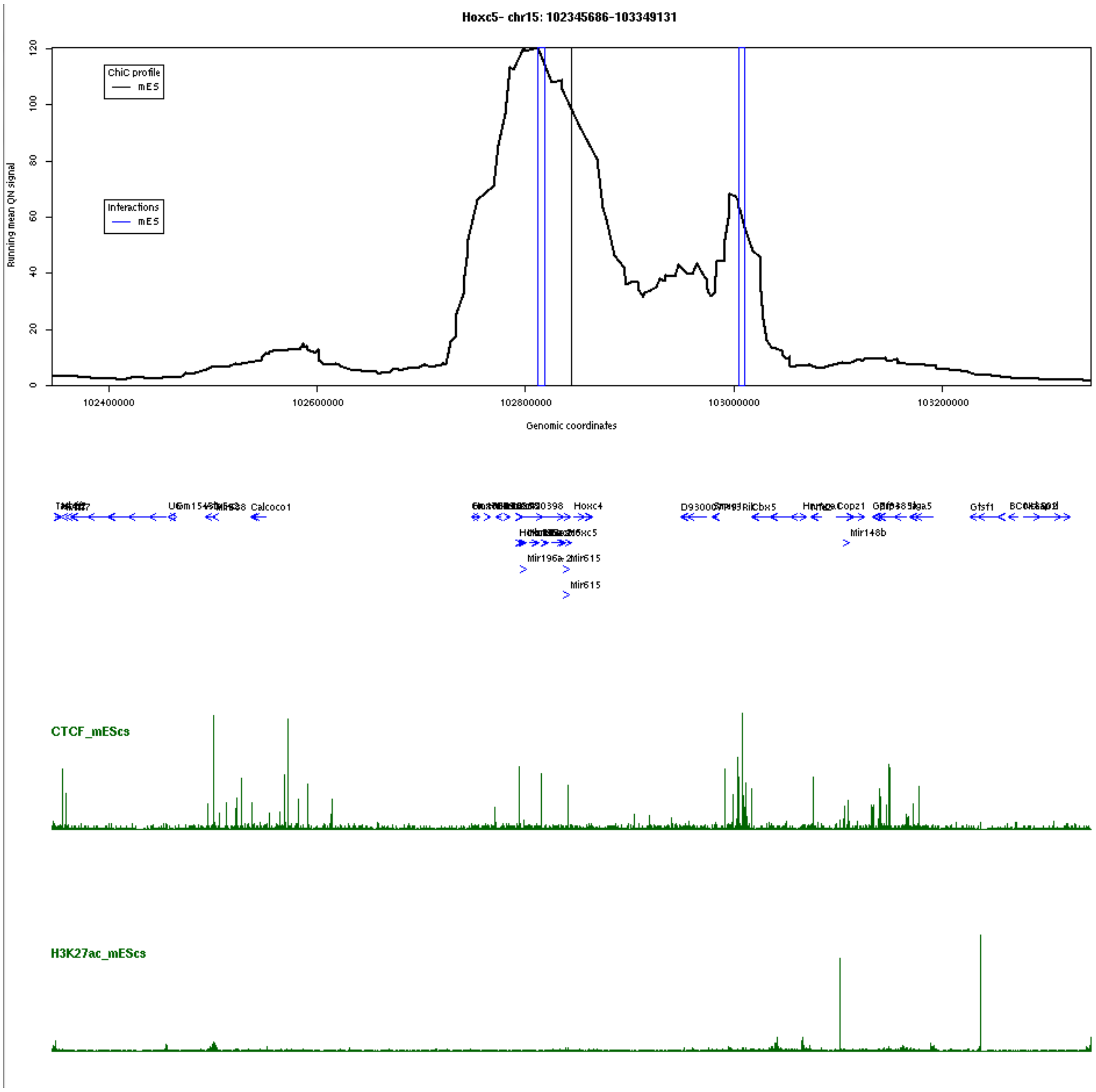

The top panel gives the CHi-C profile(s), depending on the *Conditions* settings. In all cases, the running mean (of 10 covered fragments) is plotted against the genomic coordinate. If more than one profile is plotted, they are quantile normalized to allow fairer comparison. In this case, the mean of the two replicates (named “mES” in the legend) is plotted in black. The black vertical line indicates the position of the bait, and the plot title gives the bait name and the chromosomal coordinates of the plot window. The highlighted interactions (in this case, the “mES” according to the legend, in

blue) are given as open rectangles spanning the coordinates of the non-bait fragment(s). Underneath the CHi-C panel, the positions of genes are given in blue arrows (denoting transcriptional direction), and then the selected epigenomic tracks are plotted. Using the control panel's pull-down menus and control panel, entirely new baits/plot windows can be selected, with different configurations for conditions, highlighted interactions or added epigenomic tracks, without the need to reload the data.

References

- 1. Quinlan AR, Hall IM. BEDTools: a flexible suite of utilities for comparing genomic features. Bioinformatics. 2010;266:841-2.
- 2. Schoenfelder S, Furlan-Magaril M, Mifsud B, Tavares-Cadete F, Sugar R, Javierre BM, et al. The pluripotent regulatory circuitry connecting promoters to their long-range interacting elements. Genome research. 2015;254:582-97.
- 3. Wingett S, Ewels P, Furlan-Magaril M, Nagano T, Schoenfelder S, Fraser P, et al. HiCUP: pipeline for mapping and processing Hi-C data. F1000Research. 2015;4:1310.
- 4. Cairns J, Freire-Pritchett P, Wingett SW, Varnai C, Dimond A, Plagnol V, et al. CHiCAGO: robust detection of DNA looping interactions in Capture Hi-C data. Genome biology. 2016;171:127.
- 5. Servant N, Varoquaux N, Lajoie BR, Viara E, Chen CJ, Vert JP, et al. HiC-Pro: an optimized and flexible pipeline for Hi-C data processing. Genome biology. 2015;16:259.
