# Supplementary material for: ChiCMaxima: a robust and simple pipeline for detection and visualization of chromatin looping in Capture Hi-C
Source: Genome Biol. 2019 May 22;20:102. doi: 10.1186/s13059-019-1706-3 (PMC6532271; doi:10.1186/s13059-019-1706-3)
Supplement: Supplementary file 3 — Optimizing ChiCMaxima parameters. (PDF 274 kb) [file 13059_2019_1706_MOESM3_ESM.pdf]

ChiCMaxima: a robust and simple pipeline for detection and visualization of chromatin looping in Capture Hi-C

Additional File 3: Optimizing ChiCMaxima parameters

Yousra Ben Zouari, Anne Molitor, Natalia Sikorska, Vera Pancaldi and Tom Sexton

1. Overview and formal definition of first parameters

The following parameter sweep was performed on a subset of one replicate of mES CHi-C data [1], corresponding to all the intrachromosomal bait-to-non-bait reads for 2000 randomly sampled baits (1,495,413 different potential interactions). ChiCMaxima takes each virtual 4C profile separately for the region spanning *c* bp up-and downstream of the bait, and performs a loess smoothing with span *s*. The local maxima across sliding windows of *w* fragments are initially called as interactions. Thus there are three initial parameters to tune: *w*, *s* and *c*.

2. Stringency is heavily dependent on local maximum calling window, w

With insufficient prior knowledge of "true positive" interactions within this dataset (previous 4C studies are not exhaustive enough), three metrics are used to collectively estimate the initial performance of the calling algorithm:

- Total numbers of interactions called, as an indicator of overall stringency (*specificity*, or *true negative detection rate*, in statistical terms). Alternatively, calling too few interactions implies a poor *sensitivity* (*recall* or *true positive rate*). Note that it is largely unknown how many "true" interactions there really are genome-wide; although we cannot really measure the receiver operating characteristics of the models, for the validity of future analyses we believe it is better to have greater specificity at the potential cost of sensitivity.
- Numbers of supporting sequence reads per interaction (*N*), as an indicator of the confidence that the interaction is real.
- Genomic distance of the interaction (*Distance*). It is established that shorter-range interactions have more supporting reads and are "easier" to detect [2, 3]; however famous functional chromatin loops have been detected over distances greater than a megabase [4-6]. A goal of the parameter tuning is to stringently call higher-confidence interactions without introducing excessive bias for shorter-range interactions.

Keeping *s* at 0.05 and *c* at 1.5 Mb, trying different *w* (20, 30, 50) shows that increasing *w* increases stringency: fewer interactions are called, and the called interactions have overall greater numbers of supporting reads. However, high *w* also introduces a heavy bias towards short-range interactions.

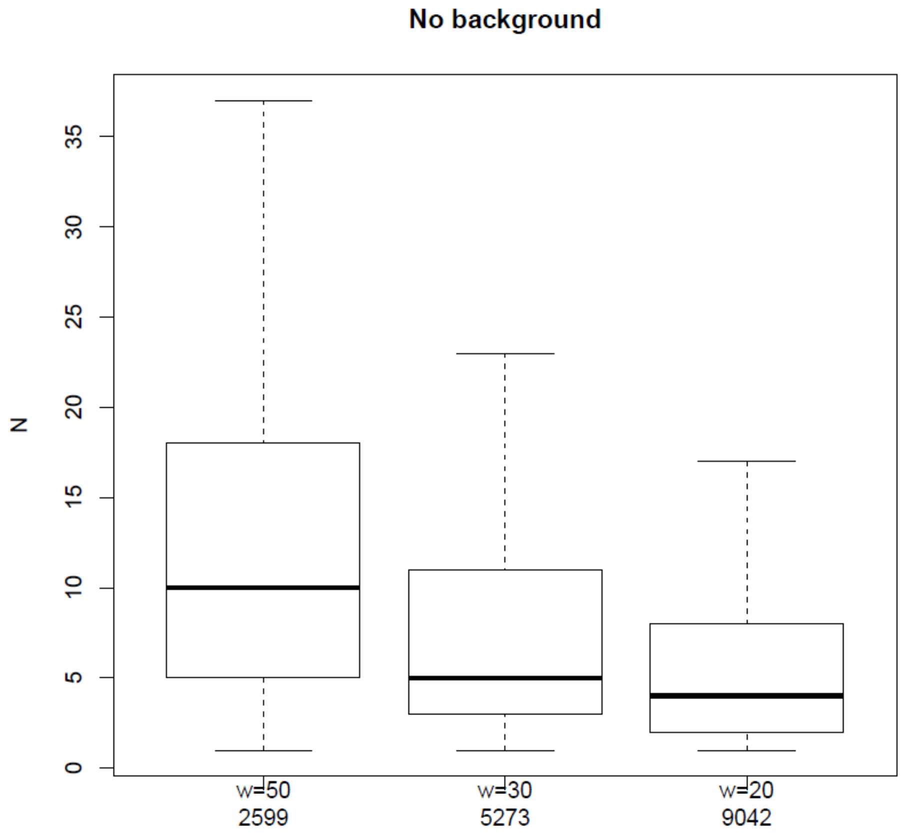

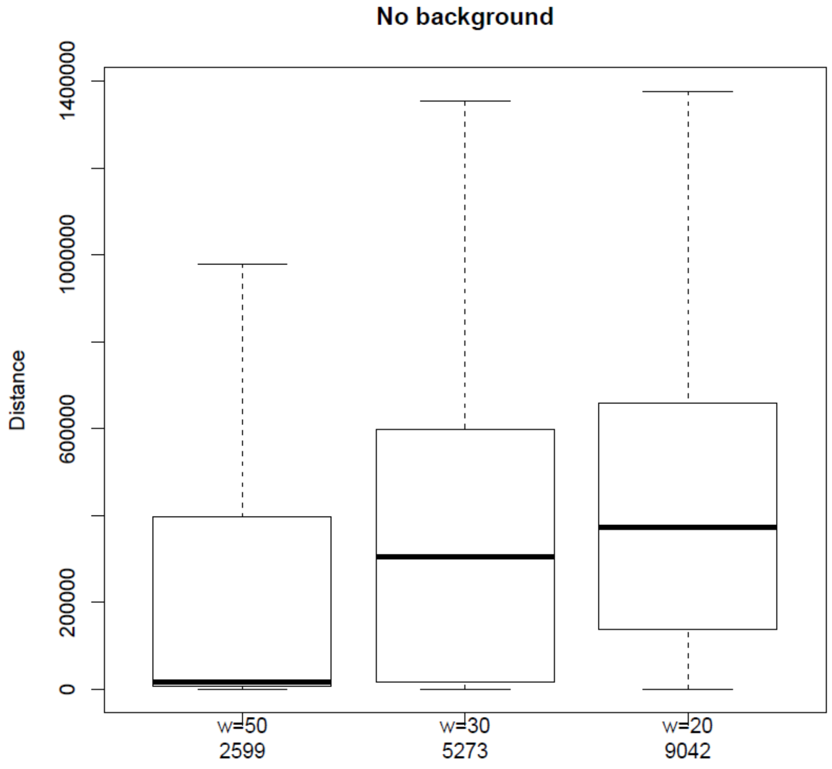

Reproduction of Additional File 1: Figure S2A,B.

3. Filtering for geometric mean offers a better compromise between stringency and distance bias than linear or polynomial fits of distance distributions

CHiCAGO estimates the relationship between genomic distance and “expected” interaction frequency by performing a cubic fit of log(N) vs log(Distance), across pooled bait datasets [2]. Trying such a fit (*Cub*) or a simpler linear fit (*LL*) of log(N) vs log(Distance) for each bait-specific dataset individually did not perform well, presumably due to overfitting sparse data. Compared to calls (*w*=20, *s*=0.05, *c*=1.5 Mb) with no filtering (*nobg*), relatively few interactions were actually filtered out as having supporting reads fewer than the background model. This is also reflected in the small changes in distribution of supporting reads per interaction (see below).

Operationally, performing the above fits requires the interactions to be stratified into fixed-width bins (in this case, 20 kb) of genomic distance of the interaction (e.g. between 0 and 20 kb, 20-40 kb, etc.), and the geometric means of these bins are used in the fits. We tested two additional filters, the first based simply on filtering for called interactions with greater read numbers than the geometric mean for its corresponding distance bin (*Geo*). The second approach, based on the overdispersed nature of sequencing data, attempted to fit a negative binomial model to this distribution of geometic mean scores (*NB*). Both models performed very similarly, conferring improved stringency over the previous models. Combining a smaller *w* with this filter appears to strike a balance between stringency of interaction calling without introducing excessive bias for shorter-range interactions.

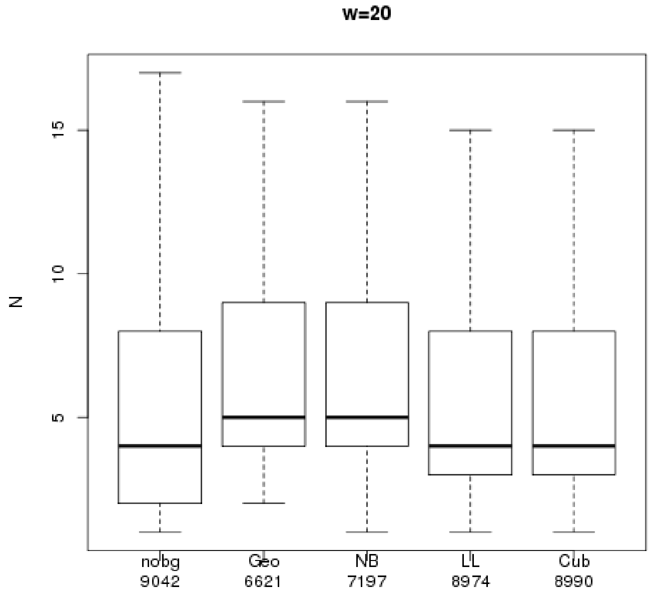

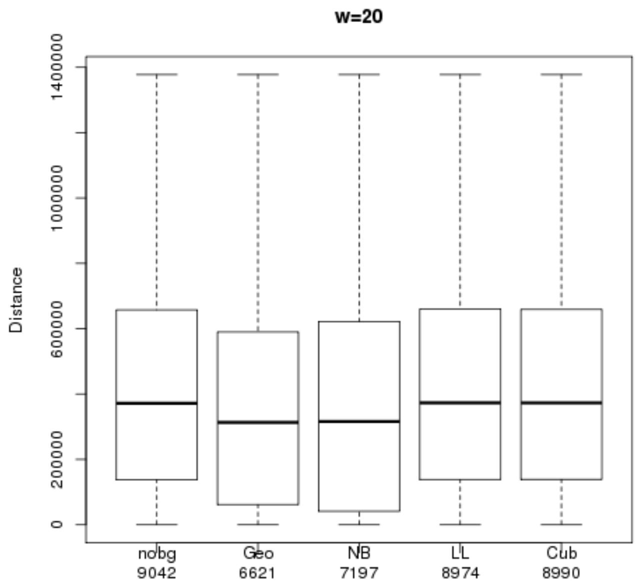

Reproduction of Additional File 1: Figure S2C,D.

Because it is simpler to implement, and the negative binomial fit fails on certain bait-specific profiles, we opted to use the geometric mean filter with smaller *w* (20) for further analyses.

4. Smaller loess span gives more consistent interaction calling

Varying the loess span, *s*, on calls does not have as large an effect as varying *w*. Smaller *s* (0.05, compared to 0.1 or 0.2) reduces the numbers of called interactions without any gain in stringency (in terms of numbers of supporting reads) and a slight increase in bias towards shorter-range interactions. We thus opted to use *s*=0.05 for further analyses.

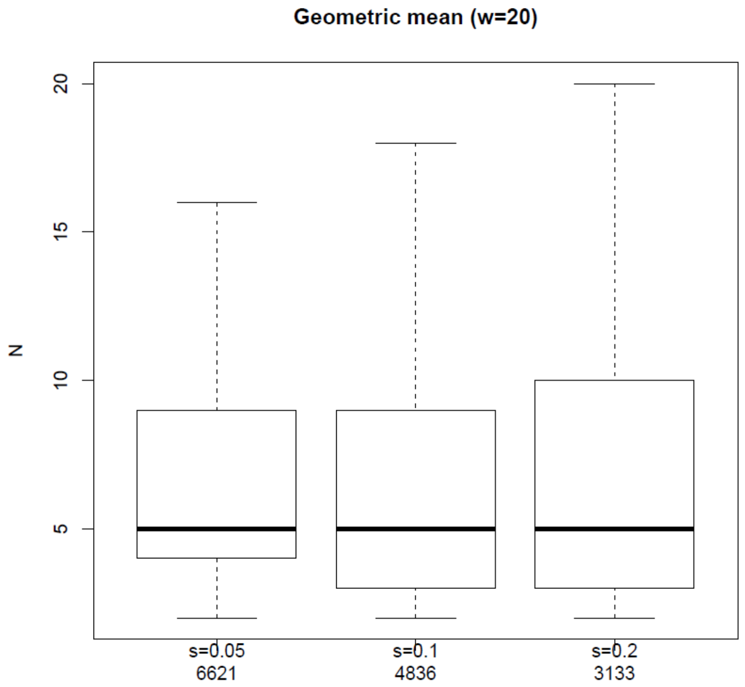

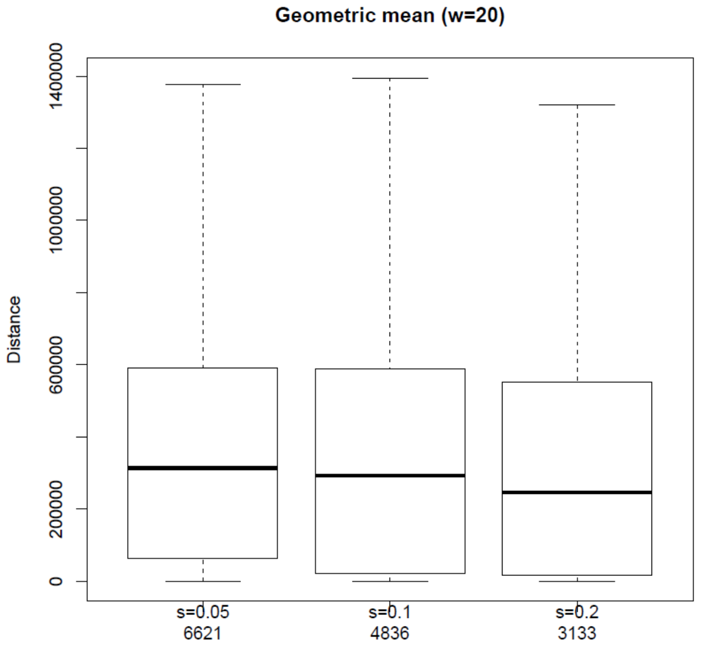

5. ChiCMaxima is relatively robust to the choice of distance bins

Whereas linear and polynomial models of CHi-C signal decay with increasing genomic separation can be interpolated to the exact interaction distance assessed, the simple geometric mean filter ascribes a single threshold for all interactions falling within a particular distance bin. There is thus a concern that the performance of the filter is heavily affected by the width of the distance bin, *b* – wider bins reduce precision of the fit, and smaller bins are more sparsely populated and more prone to overfitting. We first tested sensible limits of the *b* parameter by computing the distributions of the numbers of fragments

covered by sequences (a distance bin must be populated by at least three covered fragments for filtering to be applied). As expected, the coverage reduced with genomic distance, and this was more pronounced for smaller *b*.

**Bin width**	**Interaction distance at which median coverage < 3**
10 kb	310 kb (not good enough, considering median interaction distance is 313 kb)
20 kb (initial)	760 kb (>85% of called interactions are covered within this distance)
30 kb	1.05 Mb
50 kb	1.6 Mb

Having established that 10 kb is unworkable (at least for the sequencing depth of the test dataset), we tested the interaction calling performance for the other bin widths. Altering *b* actually makes little difference to ChiCMaxima performance. Setting *b* to 50 kb causes a slight loss of stringency (proportions of interactions with fewer supporting reads).

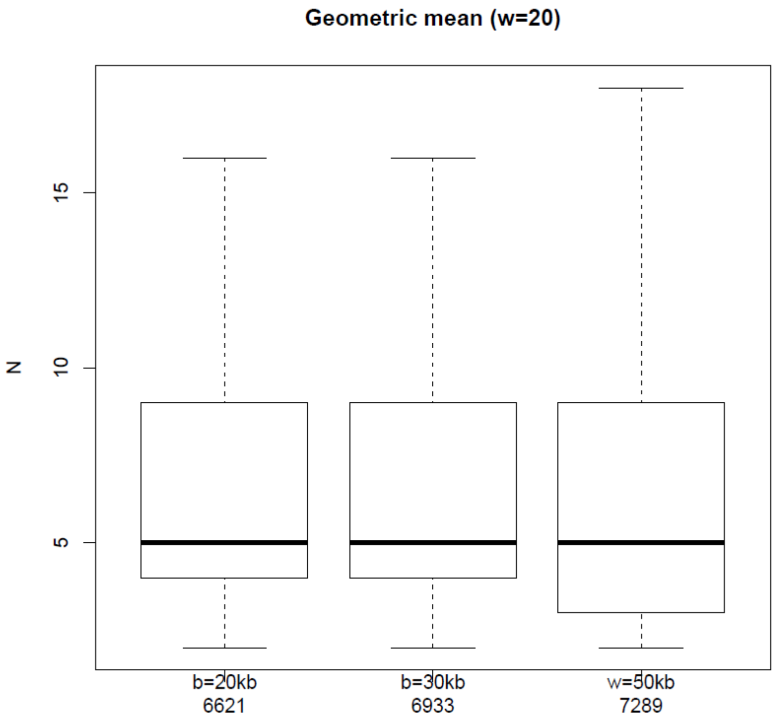

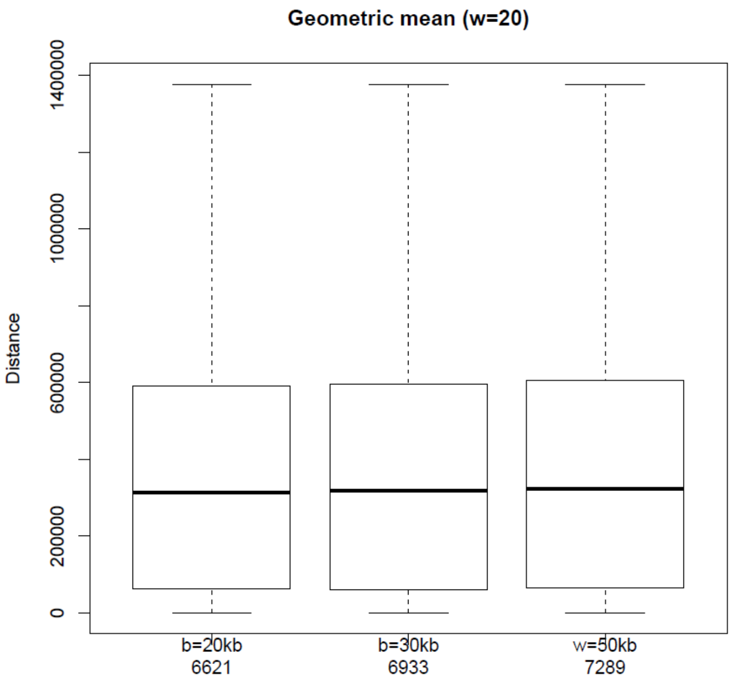

Although there is little difference in the final called interactions, we opted for *b*=30 kb, due to its improved coverage over longer-range interactions.

6. Increasing the span of ChiCMaxima calls yields limited extra interactions

The last parameter to test is *c*, the total genomic span over which local maxima are called. Initially, *c* was set to 1.5 Mb up- and downstream of the bait, the distance over which it was claimed that the background decay of interaction strength with genomic separation could be modeled in CHiCAGO [2]. To test whether this parameter is likely to prevent detection of many "real" ultra-long range interactions, ChiCMaxima was run over a span of 5 Mb up-and downstream of the bait (*w*=20, *s*=0.05, *b*=30 kb, geometric mean filter).

**Distance**	**Number of called interactions**
0-1.5 Mb (previous)	6903 (previously 6933 were called; presumably some slightly longer-range interactions were actually higher than the slightly closer ones)
1.5-2 Mb	461
2-3 Mb	505
3-5 Mb	236

As expected, called interactions get sparser with increasing distance. Based on the numbers of supporting reads, there is no obvious distance cut-off where interactions are called with appreciably less confidence.

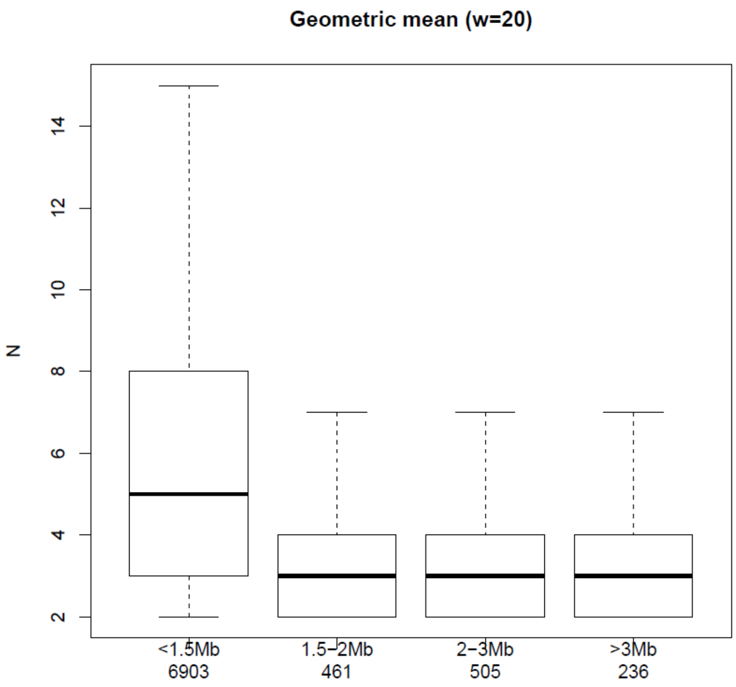

Based on the limited coverage of reads allowing geometric mean filtering at large genomic separations, and the extra computational cost in analyzing larger genomic ranges, we opted to maintain *c* at 1.5 Mb for further analyses. Importantly, called longer-range interactions are less reproducible across biological replicates, so increasing *c* actually adds little to the interactions that can be reliably called.

7. CHiCMaxima is relatively robust to most of the tunable parameters

For the bulk of this analysis, we have opted for the following conditions, which appear to offer the best compromise of stringency and limiting bias to interaction distance: *w*=20, *s*=0.05, *b*=30 kb, *c*=1.5 Mb, geometric mean filter. *A priori*, the experimental factor most likely to alter the best-performing parameters is sequencing depth, since this may allow more interactions to be called with maintained stringency on reducing *b* and increasing *c*.

Importantly, we tested quite different ChiCMaxima parameters on the full mES dataset (Additional File 1: Figure S4a), and observed in our benchmarking tests that the performance was consistently better than CHiCAGO and GOTHiC.

References

1. Schoenfelder S, Furlan-Magaril M, Mifsud B, Tavares-Cadete F, Sugar R, Javierre BM, et al. The pluripotent regulatory circuitry connecting promoters to their long-range interacting elements. Genome research. 2015;254:582-97.
2. Cairns J, Freire-Pritchett P, Wingett SW, Varnai C, Dimond A, Plagnol V, et al. CHiCAGO: robust detection of DNA looping interactions in Capture Hi-C data. Genome biology. 2016;171:127.
3. Lieberman-Aiden E, van Berkum NL, Williams L, Imakaev M, Ragoczy T, Telling A, et al. Comprehensive mapping of long-range interactions reveals folding principles of the human genome. Science. 2009;3265950:289-93.

4. Bahr C, von Paleske L, Uslu VV, Remeseiro S, Takayama N, Ng SW, et al. A Myc enhancer cluster regulates normal and leukaemic haematopoietic stem cell hierarchies. Nature. 2018;5537689:515-20.
5. Herranz D, Ambesi-Impiombato A, Palomero T, Schnell SA, Belver L, Wendorff AA, et al. A NOTCH1-driven MYC enhancer promotes T cell development, transformation and acute lymphoblastic leukemia. Nature medicine. 2014;2010:1130-7.
6. Lettice LA, Heaney SJ, Purdie LA, Li L, de Beer P, Oostra BA, et al. A long-range Shh enhancer regulates expression in the developing limb and fin and is associated with preaxial polydactyly. Human molecular genetics. 2003;1214:1725-35.
